# The avermectin, emamectin benzoate, kills gram-positive bacteria and targets the cell envelope of *Bacillus subtilis*

**DOI:** 10.1128/spectrum.00274-25

**Published:** 2025-09-02

**Authors:** Rabih Ahmad, Drew Lewis, Lou Ann Verellen, Raymond Yin, Andrew Scott, Michael Fruci

**Affiliations:** 1 London Research and Development Centre, Agriculture and Agri-Food Canada 6337, Ontario, Canada; 2 Department of Microbiology and Immunology, University of Western Ontario 6221, London, Ontario, Canada; UCI Health, Orange, California, USA

**Keywords:** avermectin, milbemycin, *Bacillus subtilis*, antibacterials, cell envelope stress response, emamectin benzoate, selamectin, *Staphylococcus aureus*

## Abstract

The avermectin family of anthelmintics is largely considered to lack antibacterial activity against gram-positive and gram-negative bacteria. Here, we screened six avermectins (ivermectin, eprinomectin, doramectin, abamectin, selamectin, and emamectin benzoate) and a structurally related milbemycin (moxidectin) for antibacterial activity against a panel of representative gram-positive and gram-negative bacteria. We report that emamectin benzoate exhibited activity against several species of gram-positive bacteria, whereas selamectin was active against *Staphylococcus aureus* and *Staphylococcus epidermidis*. Emamectin benzoate was the only avermectin with activity against hyper-permeabilized *Escherichia coli* cells. Using the model bacterium, *Bacillus subtilis*, we demonstrated that emamectin benzoate is bactericidal, causing rapid lysis and extensive cell envelope damage. To gather insight into its molecular target, several mutant selection methods were employed but failed to yield emamectin benzoate-resistant mutants. Macromolecular synthesis assays indicated that emamectin benzoate inhibited peptidoglycan synthesis. Transcriptome sequencing showed that emamectin benzoate activates the Lia and SigM cell envelope stress-responsive systems and induces the expression of peptidoglycan biosynthetic genes. Although the inactivation or activation of the Lia system did not alter the susceptibility of *B. subtilis* to emamectin benzoate, CRISPRi-mediated repression of individual essential peptidoglycan biosynthetic genes sensitized *B. subtilis* to emamectin benzoate. Emamectin benzoate synergized with cell wall-targeting antibiotics (nisin, fosfomycin, daptomycin, and tunicamycin) against *B. subtilis*. Phosphatidylglycerol antagonized the antibacterial activity of emamectin benzoate, suggesting that EMB likely interacts with membrane phospholipids to exert its effect. Altogether, avermectins exhibit anti-gram-positive activity, albeit with differing levels of potency and selectivity, and, mechanistically, emamectin benzoate targets the cell envelope.

**IMPORTANCE:**

Avermectins and the structurally related milbemycins are thought to lack antibacterial activity against gram-positive and gram-negative bacteria. Using antimicrobial susceptibility testing, we showed that avermectins and the milbemycin, moxidectin, possess anti-gram-positive activity, with the avermectin, emamectin benzoate, exhibiting the greatest spectrum of activity. Using *B. subtilis* as a model organism, we showed that emamectin benzoate is bactericidal, causes extensive cell envelope damage, and inhibits peptidoglycan synthesis. Transcriptome analysis of *B. subtilis* cells treated with emamectin benzoate showed that this avermectin activates two envelope stress-responsive systems and induces the expression of peptidoglycan biosynthetic genes, likely to counteract emamectin benzoate-mediated cell envelope damage. As some avermectins and milbemycins are approved for human and animal use, these drugs may be repurposed for the treatment of gram-positive bacterial infections. Furthermore, given the extensive use of these agents in agriculture, aquaculture, and medicine, examination of their off-target effects on various bacterial communities is warranted.

## INTRODUCTION

The avermectin family of anthelmintics is extensively used to control parasites and pests of humans, animals, and crops (1). Avermectins were discovered in the 1970s, when a group of scientists led by Dr. William Campbell of Merck & Co. in the United States tested soil-borne bacterial isolates provided by a group of scientists led by Professor Satoshi Ōmura of the Kitasato Institute in Japan for antiparasitic activity (1). Fermentation broth produced by one isolate had exceptional antiparasitic activity (1, 2) and was named *Streptomyces avermitilis* (to reflect that this bacterium facilitated the creation of a wormless “averminous” condition) (3). The active substance consisted of a mixture of eight structurally related homologues (A_1a_, A_1b_, A_2a_, A_2b_, B_1a_, B_1b_, B_2a_, and B_2b_) that differed from one another at three positions, C5, C22–23, and C25, and were collectively referred to as avermectins (2, 4, 5) (Fig. S1). Among these, both the B_1a_ and B_1b_ homologs showed the greatest activity, and since separation of these two components is complicated, the commercial product abamectin (ABM; non-proprietary name assigned) is comprised of a mixture of B_1a_ (present at ≥80%) and B_1b_ (present at ≤20%) (5–7). Further studies showed that ABM had activity against a diverse range of nematodes, insects, and mites and was subsequently developed for use in crop production and veterinary medicine (8, 9). Importantly, ABM would serve as the precursor for the semi-synthetic avermectins, including the so-called wonder drug, ivermectin (IVM) (10), as well as eprinomectin (EPM) (11), and emamectin benzoate (EMB) (12).

To date, six avermectins have been introduced into the market, each with different uses and biological properties. IVM, a hydrogenated derivative of ABM (Fig. S2), is used in both human and animal medicine to treat endo- and ecto-parasitic infections and was labeled as the first endectocide (13). In 2023, IVM was included on the World Health Organization’s list of essential medicines for its use as an intestinal anthelmintic, antifilarial, and ectoparasiticide (14). EPM is used as a broad-spectrum endectocide in cattle, including lactating dairy cows, as it was found to have a favorable milk residue profile compared with IVM (11, 15). Produced by mutational biosynthesis, doramectin (DRM) is used to control nematode and arthropod parasites in livestock (16–18). Selamectin (SEL), a derivative of DRM, was developed for companion animals and is used as an endectocide to treat heartworms, fleas, ticks, and intestinal hookworms in both cats and dogs (19). Although ABM exhibited activity against certain insects, it was ineffective against insects belonging to the order *Lepidoptera*, and this led to the development of emamectin (12). Emamectin is formulated as a salt with benzoic acid to improve thermal stability and water solubility, and the resultant compound, EMB (mixture of >90% B1a and <10% B1b), is used to control lepidopterous insects in crops and forests (20) and to control sea lice in farmed fish (21). Collectively, avermectins have improved the lives of humans and animals and have undoubtedly boosted livestock, aquaculture, and crop productivity worldwide.

All avermectins share a common pharmacophore consisting of a 16-membered macrocyclic lactone ring, to which three units are attached: a benzofuran unit (C2–C8a), a spiroketal unit (C17–C28), and a disaccharide unit (C13) (Fig. S1 and 2) (9). Modifications to the terminal saccharide and the spiroketal unit give rise to the different commercially available avermectins (22, 23). For example, substitution of the hydroxyl group at the C4” position of the terminal saccharide in ABM with a methylamino group yields EMB (Fig. S2). The anthelmintic milbemycins (e.g., moxidectin [MOX] and milbemycin oxime) are structurally related to avermectins but lack the C13 disaccharide unit (Fig. S2) (24). MOX, in particular, also contains a methoxime group at C23 and a dimethyl-butyl group at C25 (24). Milbemycins, naturally produced by *Streptomyces* species (but not *S. avermitilis*), also have excellent acaricidal and insecticidal activity and are widely used for veterinary, medical, and agricultural purposes (24–27). More recently, in 2018, the Food and Drug Administration (FDA) approved MOX for the treatment of human onchocerciasis in patients aged 12 years and older (28, 29).

Beyond their anthelmintic, insecticidal, and miticidal properties, drug repurposing studies have identified potential new uses for avermectins as they have been shown to possess antiviral (30, 31), anticancer (32), and anti-plasmodium activities (33). Although they share common structural features with macrolide antibiotics and antifungal macrocyclic polyenes, the avermectins were initially reported as lacking significant antibacterial and antifungal activity (1, 2, 23). In screening for new anti-tuberculosis treatments, Lim et al. serendipitously discovered the bactericidal activity of IVM, SEL, DRM, and MOX against multidrug and extensively drug-resistant clinical isolates of *Mycobacterium tuberculosis*, with SEL being the most potent (34). In subsequent *in vitro* studies, ABM, DRM, EMB, EPM, IVM, SEL, and milbemycins were shown to inhibit the growth of other mycobacterial species, including *Mycobacterium ulcerans* and *Mycobacterium marinum*, but with differing levels of potency (35, 36). Still, Lim et al. showed that IVM, SEL, DRM, and MOX lacked activity against representative gram-negative and gram-positive bacteria, an observation that was interpreted as avermectins being selective for mycobacteria (34). Recently, conflicting findings were reported by Folliero et al., who demonstrated the concentration-dependent bacteriostatic and bactericidal activity of SEL against clinical strains of *Staphylococcus aureus*, raising the possibility that other avermectins may possess antibacterial activity (37).

In the present study, we examined the antibacterial activity of six avermectins (ABM, IVM, EPM, DRM, SEL, and EMB) and the milbemycin, MOX, against a panel of representative gram-positive and gram-negative bacteria. EMB was the only avermectin with activity against all gram-positive bacteria examined, whereas the other avermectins and MOX exhibited varying degrees of potency and selectivity against gram-positive bacteria. Since EMB exhibited the greatest spectrum of activity, we studied this avermectin in greater detail. Using *Bacillus subtilis* as a model organism, we provide insight into the mode of action of EMB, identify its potential molecular target, and show that it does not rapidly produce resistance.

## MATERIALS AND METHODS

### Bacterial strains and growth conditions

The bacterial strains used in this study are summarized in Table 1. All bacterial strains were maintained on Luria-Bertani (LB) agar (Becton, Dickinson and Company, Sparks, MD, USA) at 37°C, unless otherwise stated. *B. subtilis* CRISPRi knockdown strains were maintained on LB agar supplemented with chloramphenicol (6 µg/mL) (38). *B. subtilis* knockout strains were maintained on LB agar supplemented with 7.5 µg/mL kanamycin (39). All overnight, liquid bacterial cultures were grown in Mueller-Hinton II (MHII) broth (MilliporeSigma, Oakville, ON, Canada) at 37°C, except for *E. faecalis* and *S. epidermidis,* which were grown in Difco Brain Heart Infusion broth (Becton, Dickinson and Company). MHII broth cultures of *B. subtilis* knockdown and knockout strains were supplemented with chloramphenicol (6 µg/mL) and kanamycin (3.5 µg/mL), respectively.

**TABLE 1:** Bacterial strains

Strain	Relevant characteristics	Source or reference
*B. subtilis* strains		
*B. subtilis* 1A1	*trpC2*	BGSC^*a*^
BKK04230	*trpC2* Δ*amj(ydaH)::kan*	(39)
BKK33100	*trpC2* Δ*liaF::kan*	(39)
BKK33110	*trpC2* Δ*liaG::kan*	(39)
BKK33120	*trpC2* Δ*liaH::kan*	(39)
BKK33130	*trpC2* Δ*liaI::kan*	(39)
BKK33080	*trpC2* Δ*liaR::kan*	(39)
BKK33090	*trpC2* Δ*liaS::kan*	(39)
BKK25000	*trpC2* Δ*pbpA::kan*	(39)
BKK34440	*trpC2* Δ*pbpE::kan*	(39)
BKK34430	*trpC2* Δ*racX::kan*	(39)
CAG74399_1	*trpC2* No-sgRNA Control Strain	(38)
BEC15220	*trpC2 lacA::Pxyl-dcas9 amyE::Pveg-sgRNA(murG*)	(38)
BEC15230	*trpC2 lacA::Pxyl-dcas9 amyE::Pveg-sgRNA(murB*)	(38)
BEC36760	*trpC2 lacA::Pxyl-dcas9 amyE::Pveg-sgRNA(murAA*)	(38)
BEC04570	*trpC2 lacA::Pxyl-dcas9 amyE::Pveg-sgRNA(murF*)	(38)
BEC01780	*trpC2 lacA::Pxyl-dcas9 amyE::Pveg-sgRNA(glmS*)	(38)
BEC00500	*trpC2 lacA::Pxyl-dcas9 amyE::Pveg-sgRNA(glmU*)	(38)
BEC15200	*trpC2 lacA::Pxyl-dcas9 amyE::Pveg-sgRNA(murD*)	(38)
BEC01770	*trpC2 lacA::Pxyl-dcas9 amyE::Pveg-sgRNA(glmM*)	(38)
BEC16530	*trpC2 lacA::Pxyl-dcas9 amyE::Pveg-sgRNA(uppS*)	(38)
BEC29790	*trpC2 lacA::Pxyl-dcas9 amyE::Pveg-sgRNA(murC*)	(38)
BEC15190	*trpC2 lacA::Pxyl-dcas9 amyE::Pveg-sgRNA(mraY*)	(38)
BEC15180	*trpC2 lacA::Pxyl-dcas9 amyE::Pveg-sgRNA(murE*)	(38)
*E. coli* strains		
*E. coli* BW25113	*E. coli* K-12 BW25113 wild type: Δ(*araD-araB*)567 Δ*lacZ*4787(::*rrnB*-3) *rph*-1 Δ(*rhaDrhaB*)568 *hsdR*514	Keio Collection
*E. coli* BW25113Δ*bamB*Δ*tolC*	BW25113 in which *bamB* and *tolC* were deleted to create a hyperpermeable, efflux-deficient strain	(40)
Other strains		
*Staphylococcus aureus subsp. aureus* Rosenbach (ATCC 25923)	Quality control strain, clinical isolate	ATCC
*Staphylococcus epidermidis* (Winslow and Winslow) Evans (ATCC 12228)	Quality control strain	ATCC
*Enterococcus faecalis* (Andrewes and Horder) Schleifer and Kilpper-Balzz (ATCC 29212)	Quality control strain, isolated from urine	ATCC
*Aeromonas hydrophila* (Chester) Stanier (ATCC 7966)	Quality control strain, isolated from tin of milk	ATCC
*Salmonella enterica* subsp. *enterica* (ex Kauffmann and Edwards) Le Minor and Popoff serovar Typhimurium (ATCC 14028)	Quality control strain, isolated from pooled heart and liver tissues of four-week-old chickens	ATCC
*Klebsiella quasipneumoniae* Brisse et al. (ATCC 700603)	Quality control strain, isolated from urine of hospitalized patient, produces extended-spectrum β-lactamase (SHV-18)	ATCC
*Pseudomonas aeruginosa* PAO1	PAO1 prototroph	Poole, K
*Yersinia enterocolitica* subsp. *enterocolitica* (Schleifstein and Coleman) Frederiksen (ATCC 23715)	Applications in enteric disease research and media testing	ATCC

^*a*^ BGSC, Bacillus Genetic Stock Centre.

### Antibiotics, avermectins, and moxidectin

The stock concentration, solvent, and source of avermectins, MOX, and antibiotics used in this study are as follows: EMB (Toronto Research Chemicals, Vaughan, ON, Canada), 50 mg/mL in ethanol; ABM (AK Scientific, Union City, CA, USA), 20 mg/mL in ethanol; DRM (AK Scientific), 40 mg/mL in DMSO; EPM (Sigma-Aldrich, Oakville, ON, Canada), 20 mg/mL in DMSO; IVM (AK Scientific), 40 mg/mL in DMSO; SEL (Sigma-Aldrich), 40 mg/mL in DMSO; and MOX (AK Scientific), 20 mg/mL in ethanol; erythromycin (MP Biomedicals, Solon, OH, USA), 1 mg/mL in water; bacitracin-zinc (AK Scientific), 100 mg/mL in 0.02 N HCl; chloramphenicol (Sigma-Aldrich), 50 mg/mL in ethanol; daptomycin (AK Scientific), 5 mg/mL in methanol; fosfomycin disodium (TCI America, Portland, OR, USA), 50 mg/mL in water; nisin (Sigma-Aldrich), 50 mg/mL in 0.02N HCl; tetracycline (Bio Basic, Markham, ON, Canada), 50 mg/mL in methanol; vancomycin (AK Scientific), 50 mg/mL in water; tunicamycin (Sigma-Aldrich), 5 mg/mL in DMSO; ciprofloxacin (Bio Basic), 10 mg/mL water; rifampicin (AK Scientific), 10 mg/mL in DMSO; kanamycin (Sigma-Aldrich), 50 mg/mL in water; and triclosan (MilliporeSigma), 50 mg/mL in ethanol.

### Antimicrobial susceptibility testing

The susceptibility of the various bacterial strains to antimicrobial agents was determined using the 2-fold serial microtiter broth dilution method as previously described (41), with the exception that MHII broth was used. For the CRISPRi knockdown strains, susceptibility testing was performed using MHII broth supplemented with 0.001%, 0.005%, or 0.01% xylose (Bio Basic). Unless otherwise stated, the minimum inhibitory concentration (MIC) was recorded as the lowest concentration of drug that inhibited visible growth after 18 hours of incubation at 37°C. For antimicrobial susceptibility testing of permeabilized *E. coli* cells, EDTA was added to MHII broth to a final concentration of 0.1 mM, 1 mM, or 10 mM. The optical density at 600 nm (OD_600_) of growth in the wells of the microtiter plates was determined using a BioTek Synergy HTX Multi-Mode microplate reader (BioTek, Winooski, VT, USA).

### Time-kill assay

Time-kill assays were performed as described by the CLSI guidelines (42). Overnight cultures of *B. subtilis* 1A1 or *S. aureus* ATCC 25923 were subcultured (1:49 dilution) in sterile MHII broth or LB broth, respectively, at 37°C with shaking at 225 rpm until an OD_600_ of ~0.5. *B. subtilis* or *S. aureus* cells were aliquoted into five separate flasks (10 mL each) to a final cell density of ~4 × 10^5^ CFU/mL. *B. subtilis* cells were treated as follows: no drug control (growth control), vehicle control (ethanol only; ethanol was added at the same concentration as cells exposed to 32 µg/mL EMB), and EMB at 8 µg/mL (1/4× MIC), 16 µg/mL (1/2× MIC), or 32 µg/mL (1× MIC). *S. aureus* cells were treated as follows: no drug control (growth control), vehicle control (DMSO only; DMSO was added at the same concentration as cells exposed to 32 µg/mL SEL), and SEL at 8 µg/mL (1/2× MIC), 16 µg/mL (1× MIC), or 32 µg/mL (2× MIC). A 1 mL aliquot of cells was removed at time 0, 2, 4, 6, and 24 h post-treatment, centrifuged at 13,000 rpm for 3 min, reconstituted with 1 mL of MHII or LB broth, and subsequently serially diluted in MHII broth. One hundred microliters of each serial dilution was plated, each in technical duplicate, on MHII agar plates for the determination of viable counts. Total bacterial CFU/mL (log_10_ CFU/mL) was determined after 20 h of incubation at 37°C. Only the lowest dilution with 20–300 CFUs was included in analyses. A bactericidal effect was defined by a ≥ 3 log_10_ (99.9% killing) decrease in CFU/mL at the 6-h time point relative to the initial inoculum at t = 0 h.

### Cell lysis monitoring

To monitor for cell lysis, overnight-grown *B. subtilis* 1A1 cells were subcultured in a similar manner as described for the time-kill assay. Once the subculture reached an OD_600_ of 0.5–0.6, the culture was divided into five separate flasks (10 mL each), and cultures were treated as follows: no drug control (growth control), vehicle control (ethanol only, added at the same concentration used as cells exposed to 32 µg/mL EMB), and EMB at concentrations of 8 µg/mL (1/4× MIC), 16 µg/mL (1/2× MIC), or 32 µg/mL (1× MIC). Cultures were then incubated at 37°C with shaking at 225 rpm. At 0, 15, 30, and 60 min after treatment, 800 µL of culture was removed and the OD_600_ was determined using a NanoDrop OneC spectrophotometer (Thermo Fisher Scientific, Toronto, ON, Canada). For photographs of lysed cell cultures, 10 mL cultures of *B. subtilis* at an OD_600_ of 0.5–0.6 were treated with 1× MIC of EMB or AMP (0.125 µg/mL) for 60 min, after which 5 mL of culture was added to sterile, clear polystyrene culture tubes and photographed. Each experiment was performed in at least three biological replicates.

### Selection of EMB-resistant mutants

Three selection methods were employed in an attempt to recover EMB-resistant *B. subtilis* mutants. (i) Overnight cultures of *B. subtilis* cells diluted (1:49) were serially passaged over a 15-day period in MHII broth containing subinhibitory concentrations of EMB (2, 4, 8, or 16 µg/mL). On the 15th day, the cells were plated on MHII agar containing the MIC of EMB (32 µg/mL) and incubated at 37°C for 14 days. In all instances, no resistant colonies were recovered. Experiments were performed with at least three biological replicates. (ii) To isolate spontaneously resistant mutants, an overnight culture of *B. subtilis* was serially diluted and spread plated onto MHII agar plates containing EMB at 32 µg/mL (1× MIC) and incubated for 21 days. Again, no spontaneously resistant mutants were observed. Experiments were performed with at least three biological replicates, and each serial dilution was plated in technical duplicate. (iii) To increase the chances of selecting for EMB-resistant *B. subtilis* mutants, the evolutionary ramp method in microtiter plates and checkerboard format was employed in a similar manner as previously outlined (43). A total of 90 individual *B. subtilis* colonies were cultured overnight each in 200 µL of MHII broth supplemented with 1/8× MIC of EMB (4 µg/mL) in 96-well microtiter plates (Corning Costar, Sigma-Aldrich), at 37°C, with shaking at 200 rpm. Overnight cultures were then passaged on a daily basis starting at 1/8× MIC of EMB (4 µg/mL), with drug concentration doubling each day until reaching the MIC of EMB. An unexposed control, consisting of 30 individual colonies of *B. subtilis*, was passaged on a daily basis for the same duration as the treatment group. Although *B. subtilis* grew at sub-MIC levels of EMB, there was no visible growth of *B. subtilis* following treatment with the MIC of EMB after 24 h. The 90 populations exposed to 1× MIC of EMB were spot plated onto MHII agar plates supplemented with 1× MIC of EMB and incubated for 14 days. No colonies were observed after 14 days of incubation.

### RNA sequencing

*B. subtilis* 1A1 cells grown overnight in MHII broth at 37°C were subcultured (1:49) in the same medium and incubated at 37°C, shaking at 200 rpm, until cultures reached an OD_600_ of 0.5–0.6. The culture was divided into two separate flasks (10 mL each) and treated with either EMB [8 µg/mL (1/4× MIC) final concentration] or ethanol (vehicle control; ethanol was added at the same concentration used in the 1/4× MIC of EMB-exposed cells) for 30 min prior to harvesting the cells. Total RNA was isolated from four biological replicates using the High Pure RNA Isolation Kit (Roche Diagnostics, Mississauga, ON, Canada) and a protocol outlined by the manufacturer. Remaining traces of DNA in isolated RNA samples were eliminated with Turbo DNAase (Thermo Fisher Scientific) for 1 h at 37°C. DNA-free RNA was confirmed by the failure to PCR amplify the *recA* gene using the *recA* For. (5′- GTATACGCGCAAAAGCTCGG-3′) and *recA* Rev. (5′-ATGTCAACCTCGGCTGTACG-3′) primers, along with GoTaq DNA polymerase kit and a PCR protocol outlined by the manufacturer. Concentration and purity of RNA was determined using a Nanodrop OneC Spectrophotometer (Thermo Fisher Scientific). RNA processing and sequencing were performed at SeqCenter (Pittsburgh, PA, USA). Briefly, samples were again DNAse-treated with Invitrogen DNAse. Library preparation was performed using Illumina’s Stranded Total RNA Prep Ligation with Ribo-Zero Plus kit and 10 bp unique dual indices (UDI). RNA was sequenced on a NovaSeq X Plus, producing paired-end 150 bp reads. Demultiplexing, quality control, and adapter trimming were performed with bcl-convert (v4.1.5). Reads were mapped with HISTAT2 (version 2.2.0). Read counts were imported into R (version 4.0.2) and normalized to the Trimmed Mean of M values (TMM) algorithm using edgeR (version 1.14.5). Values were subsequently converted to counts per million (CPM), and differential expression analysis was performed using edgeR glmQLFTest. The volcano plot was created with GraphPad Prism v9.5.0 (La Jolla, CA, USA). Gene ontology (GO) enrichment of significantly upregulated genes was analyzed using the PANTHER overrepresentation test, with *B. subtilis* as the reference list and the GO biological process complete data set (44–46). GO terms were analyzed using Fisher’s exact tests, and the results were filtered for significance with an FDR of <0.05.

### Quantitative real-time PCR

*B. subtilis* cells were cultured, and total RNA was isolated as described above. Reverse transcription of DNA-free RNA, confirmed with PCR using the *recA* primer set (see above), into cDNA was carried out in a 10 µL mixture containing 500 ng of RNA, 2 µL of the 5× iScript reaction mix, and 0.5 µL of iScript reverse transcriptase (Bio-Rad, Mississauga, ON, Canada). The reaction mixture was incubated in a thermocycler for 5 min at 25°C, followed by 20 min at 46°C and 1 min at 95°C. Relative quantification of target genes was conducted using a Bio-Rad, CFX96 Real-Time PCR instrument (Bio-Rad) with Bio-Rad CFX Maestro software version 3.0. PCR amplification reactions were performed in 20 µL reaction volumes each containing 10 µL of SsoFast EvaGreen Supermix, 500 nM each of forward and reverse primers per target gene, and 5 µL of 1:24 diluted cDNA. The primers used in quantitative real-time PCR (qRT-PCR), amplification efficiencies, and correlation coefficients are described in Table 2. The reaction mixtures were heated at 95°C for 30 s (initial denaturation), followed by 40 cycles of 5 s at 95°C and 5 s at 60°C. At the end of 40 cycles, a melt curve was obtained with an initial 10 s denaturation step at 95°C, followed by 5 s incubations of 0.5°C increments between 65°C and 95°C to ensure the presence of a single amplicon. Target gene expression levels were normalized to two reference genes, *gyrA* and *gatB*. Biological triplicates and technical triplicates were performed for all samples. To confirm the absence of DNA contamination, no-template controls were performed in technical triplicates for all primer sets employed. Oligonucleotide synthesis was conducted by Integrated DNA Technologies (Coralville, IA, USA).

**TABLE 2:** Quantitative real-time PCR primer sets

Primer	Oligonucleotide sequence (5′−3′)	Amplicon size (bp)	Amplification efficiencies (%)	Correlation coefficients	Source
liaF For.	TATCGGAATAGGCGATCTGCTG	102	103.7	0.994	This study
liaF Rev.	ATATACATGACGGAGCCAAGCC				This study
liaR For.	TATTATCCAGGCTGCGCCAC	101	109.2	0.990	This study
liaR Rev.	TGTCTTTCCTTCTGCGATCAGG				This study
liaH For.	GGAGAATCCAAAGGTGATGCTG	100	100.6	0.999	This study
liaH Rev.	ATAGGCAATCGTGTGCTGTTTC				This study
amj For.	TCCGTTCTTGCTGATGATGTGG	87	100.8	0.996	This study
amj Rev.	CACCCTTGATGTGACCATTGTG				This study
murG For.	ATGGTCACAAAGCCGTTTCTTC	104	102.4	0.999	This study
murG Rev.	AGAGCAGTAATTTCGGCAATCG				This study
gyrA For.	TCCCGAATCTGCTCGTGAAC	71	104.1	0.999	This study
gyrA Rev.	CTGGTGCGGAGGAATGTTTG				This study
gatB For.	GCGACGCTGAGAAGATTGTG	97	101.9	0.998	This study
gatB Rev.	GGATTGTTGTCAAGCGCCTC				This study

### Growth curve assay

Overnight cultures of the *B. subtilis* knockdown strains were diluted to an OD_600_ of 0.1 with MHII broth supplemented, when necessary, with 0.001% (wt/vol), 0.005% (wt/vol), and 0.01% (wt/vol) xylose. Bacterial cells were further diluted in MHII broth supplemented, when necessary, with the appropriate concentration of xylose to reach a final OD_600_ of ~0.01. The OD_600_ was monitored every 20 min for 12 h, with a Biotek Synergy HTX Multi-Mode Micro Plate Reader set at 37°C with mild shaking. Media-only controls were conducted to ensure the absence of contamination and to monitor background absorbance at 600 nm. OD_600_ values were baseline-corrected by subtracting background absorbance at 600 nm of the uninoculated media from the absorbance at 600 nm of the inoculated media.

### Checkerboard assay

Checkerboard assays were conducted as previously outlined (47), with the exception that MHII broth was used. To quantify the interaction of EMB with select antibiotics, the fractional inhibitory concentration index (FICI) value was determined as follows: A/MIC_A_ + B/MIC_B_= FIC_A_ + FIC_B_ =FICI, where MIC_A_ and MIC_B_ are the MIC of drug A and B alone, and A and B are the concentrations inhibiting bacterial growth in combination. A FICI of ≤0.5 was defined as synergistic, and a FICI range of >0.5–4 was defined as nonsynergistic.

### Macromolecular synthesis assay

The macromolecular synthesis assay in cell culture tubes was performed using a previously described protocol (48). Briefly, overnight cultures of *B. subtilis* grown in MHII broth were subcultured (1:49) in the same medium until an OD_600_ of ~0.4–0.5 was reached. Cells were pelleted by centrifugation for 10 min at 4,000 × *g* at room temperature, and the supernatant was decanted. Cells were resuspended in prewarmed M5T medium [M9 salts medium (Na_2_HPO_4_ ·7H_2_O, 12.8 g/L; KH_2_PO_4_, 3.0 g/L; NaCl, 0.5 g/L; NH_4_Cl, 1 g/L)], supplemented with 5% tryptic soy broth (TSB), 0.4% glucose, 2 mM MgSO_4_, and 0.1 mM CaCl_2_] to a final OD_600_ of 0.2. The radiolabeled precursors [5,6-^3^H]-uridine (250 µCi), [3,4,5,-^3^H]-leucine (250 µCi), [methyl-^3^H]-thymidine (250 µCi), [6-^3^H]-*N*-acetyl-D-glucosamine (50 µCi), [1-^14^C]-acetic acid (50 µCi) obtained from American Radiolabeled Chemicals (St. Louis, MO, USA) were used to measure RNA, protein, DNA, PG, and fatty acid synthesis, respectively, and were added to a final concentration of 0.1 µCi/mL to 14 mL cell culture tubes containing 2 mL of cell suspension. EMB was added to a final concentration of 1/4× or 1/2× MIC to the culture tubes. The positive control antibiotics vancomycin, triclosan, rifampicin, and chloramphenicol were added at 4× MIC, whereas ciprofloxacin was added at 8× MIC. Cell-free, compound-free blank controls and untreated controls were run in parallel. After 20 min of incubation at 37°C with shaking at 225 rpm, 0.2 mL aliquots were removed, added to 2 mL of ice-cold 10% trichloroacetic acid (TCA), and incubated on ice for 1 h to allow radiolabeled material to precipitate. Radiolabeled precipitates (2 mL) were then loaded onto Whatman 47 mm GF/C glass microfibre filters pretreated with 2 mL of 10% TCA. Filters were washed twice with 10 mL each of 5% TCA and 75% ethanol. Filters were removed, dried, and counted using Universol liquid scintillation cocktail (MP Biomedicals) and a Perkin Elmer Tri-Carb 2900TR liquid scintillation analyzer. Radiolabel-free cell growth controls were processed in parallel to ensure OD_600_ remained comparable between no drug, antibiotic, and EMB-treated cells.

### Antagonization assays

Antagonization of the antibacterial activity of EMB by potential cell wall and lipid molecules was performed in microtiter plates. EMB (1× MIC) was preincubated with purified antagonists [Ala-D-γ-Glu-Lys-D-Ala-D-Ala, *N*-acetyl-D-glucosamine, *N*-acetylmuramic acid, and peptidoglycan from *B. subtilis* were obtained from MilliporeSigma; phosphatidylglycerol (egg), undecaprenyl-monophosphate diammonium salt, and undecaprenyl-diphosphate triammonium salt were obtained from Larodan Research Grade Lipids (Solna, Sweden)], ranging from 0.5-fold to 10-fold excess (wt/vol) with respect to EMB. Bacterial cells (final inoculum of ~5 × 10^5^ CFU/mL) were mixed with EMB alone or EMB and antagonists in the wells of a microtitere plate. Wells were visually examined for growth or no growth after incubation at 37°C for 20 h. Experiments were performed with at least three biological replicates each in technical duplicates.

### Transmission electron microscopy

Mid-logarithmic phase (OD_600_ of 0.5-0.6) MHII-broth subcultures of *B. subtilis* were treated with either 1× MIC of EMB or vehicle control (ethanol) for 30 min. Samples (1 mL) were collected and pelleted by centrifugation for 2 min at 12,000 × *g*. Bacterial pellets were then resuspended with sterile 1× HEPES-buffered saline (HBS, 10 mM HEPES buffer, and 100 mM NaCl, pH 7.1) and then centrifuged for 2 min at 12,000 × *g*. Cells were then resuspended and fixed with 2.5% paraformaldehyde in HBS for 30 min on ice. Cells were then pelleted by centrifugation, washed once with HBS, and resuspended in 50 µL of HBS. Fixed cells were deposited onto transmission electron microscopy (TEM) 400-mesh Formvar/carbon copper grids (Electron Microscopy Sciences, Hatfield, PA, USA) and incubated at room temperature for 5 min. Excess cells were removed by wicking with filter paper. Samples were then stained by placing a drop of 1% (wt/vol) uranyl acetate in HBS onto the samples for 20 s, and excess stain was removed by wicking with a filter paper. Grids were then washed with a drop of sterile water and subsequently air-dried overnight. Bright field images were taken with a Jeol JEM-1400Flash electron microscope (Jeol, Peabody, MA, USA) equipped with a NANOSPRT15 camera at an accelerating voltage of 80 kV.

### Statistical analysis

Each experiment was performed in at least three biological replicates, unless otherwise noted. Graphing and data analysis were conducted using GraphPad Prism v9.5.0. Specific statistical tests are described in the figure captions.

## RESULTS

### Avermectins and moxidectin exhibit antibacterial activity

The antibacterial activity of six avermectins (ABM, DRM, EPM, IVM, EMB, and SEL) and MOX against several gram-positive bacteria was examined using the broth microdilution assay with MHII broth. Given the poor water solubility of avermectins and MOX, mirror plates, without bacteria but with the drug, served as precipitate controls. In MHII broth, SEL, ABM, DRM, EPM, and MOX precipitated at ≥32 µg/mL, whereas EMB and IVM precipitated at ≥128 µg/mL and ≥16 µg/mL, respectively. At soluble concentrations, EMB exhibited activity against all gram-positive bacteria examined, whereas SEL exhibited activity against *Staphylococcus epidermidis* (Table 3). Several avermectins and MOX visibly reduced the growth of either *Enterococcus faecalis, S. epidermidis,* or *S. aureus* as drug concentrations increased, even at concentrations in which precipitate formed in the mirror plates (Fig. S3). All avermectins and MOX hindered the growth of *E. faecalis* as drug concentrations increased, with IVM, SEL, and MOX being the most potent. Growth of *S. epidermidis* was reduced at 32 µg/mL of ABM and MOX, whereas the growth of *S. aureus* was substantially decreased in the presence of 16 µg/mL of SEL and ABM. SEL-mediated inhibition of *S. aureus* was surprising, given that Lim et al. previously reported the SEL MIC_90_ for the same strain of *S. aureus* as >256 µg/mL in LB broth (34). As growth media can influence MIC values (49), we repeated susceptibility testing of SEL with *S. aureus* in LB broth. SEL inhibited the growth of *S. aureus* at 16 µg/mL, and time-kill curves demonstrated bacteriostatic activity of SEL against *S. aureus* (Fig. S4). Furthermore, SEL precipitated in LB broth at concentrations ranging from 32 µg/mL to 256 µg/mL, thus challenging the accuracy of the previously reported SEL MIC of >256 µg/mL for *S. aureus* (34). Finally, and unlike the other gram-positive bacteria examined, there was no visible reduction in *B. subtilis* growth when exposed to increasing concentrations of DRM, IVM, EPM, ABM, SEL, and MOX (Fig. S3).

**TABLE 3:** Susceptibility of gram-positive and gram-negative bacteria to avermectins and MOX in MHII broth

	MIC (µg/mL)^a^						
	EMB	ABM	DRM	EPM	IVM	SEL	MOX
Gram-positive							
*Bacillus subtilis* 1A1	32	>16	>16	>16	>8	>16	>16
*Staphylococcus aureus*	16	>16	>16	>16	>8	>16^b^	>16
*Staphylococcus epidermidis*	32	>16	>16	>16	>8	16	>16
*Enterococcus faecalis*	32	>16	>16	>16	>8	>16^b^	>16
Gram-negative							
*Escherichia coli* BW25113	>64	>16	>16	>16	>8	>16	>16
*Aeromonas hydrophila*	>64	>16	>16	>16	>8	>16	>16
*Salmonella enterica* serovar Typhimurium	>64	>16	>16	>16	>8	>16	>16
*Klebsiella quasipneumoniae*	>64	>16	>16	>16	>8	>16	>16
*Pseudomonas aeruginosa* PAO1	>64	>16	>16	>16	>8	>16	>16
*Yersinia enterocolitica*	>64	>16	>16	>16	>8	>16	>16

^a^ EMB, emamectin benzoate; ABM, abamectin; DRM, doramectin; EPM, eprinomectin; IVM, ivermectin; SEL, selamectin; MOX, moxidectin. For all strains evaluated, a minimum of three biological replicates were performed, each in technical duplicates. Since SEL, ABM, DRM, EPM, and MOX precipitated at ≥32 µg/mL, while EMB and IVM precipitated at ≥128 µg/mL and ≥16 µg/mL, respectively, the inhibitory activity at drug concentrations that visibly precipitated could not be accurately determined.

^b^ A major reduction in visible growth of *S. aureus* and *E. faecalis* was observed at 16 µg/mL of SEL. Since growth was not completely inhibited at this concentration, the MIC for SEL was recorded as >16 µg/mL.

It has previously been shown (34), and reconfirmed here (Table 3) that avermectins and MOX lack activity against gram-negative bacteria. Since the outer membrane of gram-negative bacteria prevents many antibiotics from reaching their intracellular targets (50), the activity of avermectins and MOX against membrane-permeabilized *E. coli* cells was determined. Permeabilization of the outer membrane of wild-type (WT) *E. coli* BW25113 by EDTA at 10 mM, but not 0.1 mM or 1 mM, sensitized cells to EMB (Table 4). Next, we assessed if the hyperpermeable and efflux-pump-deficient *bamB*/*tolC* double-deletion strain of *E. coli* BW25113, with enhanced uptake of small molecules (51), demonstrated sensitivity to EMB. Deletion of *bamB*/*tolC* increased the sensitivity of *E. coli* to EMB relative to WT *E. coli*, but only in the presence of 1 mM EDTA (10 mM EDTA alone inhibited the growth of the double mutant). Permeabilization of *E. coli* cells with EDTA and/or deletion of *bamB*/*tolC* did not alter the susceptibility of *E. coli* to all other avermectins or MOX. These results suggest that the molecular target of EMB is conserved among gram-positive and gram-negative bacteria. Since EMB had the greatest spectrum of activity, EMB was studied in greater detail using the model organism *B. subtilis* 1A1.

**TABLE 4:** Impact of EDTA addition on the susceptibility of wild-type and hyperpermeable strains of *E. coli* to avermectins and MOX

Strain	Drug	MIC (μg/mL)^a^			
		EDTA (mM)^b^			
		0	0.1	1	10
*E. coli* BW25113	EMB	>64	>64	>64	32
*E. coli* BW25113 *bamB*Δ*tolC*	EMB	>64	>64	16	_—^c^_
*E. coli* BW25113	ABM	>16	>16	>16	>16
*E. coli* BW25113 *bamB*Δ*tolC*	ABM	>16	>16	>16	_—^c^_
*E. coli* BW25113	DRM	>16	>16	>16	>16
*E. coli* BW25113 *bamB*Δ*tolC*	DRM	>16	>16	>16	_—^c^_
*E. coli* BW25113	EPM	>16	>16	>16	>16
*E. coli* BW25113 *bamB*Δ*tolC*	EPM	>16	>16	>16	_—^c^_
*E. coli* BW25113	IVM	>8	>8	>8	>8
*E. coli* BW25113 *bamB*Δ*tolC*	IVM	>8	>8	>8	_—^c^_
*E. coli* BW25113	SEL	>16	>16	>16	>16
*E. coli* BW25113 *bamB*Δ*tolC*	SEL	>16	>16	>16	_—^c^_
*E. coli* BW25113	MOX	>16	>16	>16	>16
*E. coli* BW25113 *bamB*Δ*tolC*	MOX	>16	>16	>16	_—^c^_

^a^ EMB, emamectin benzoate; ABM, abamectin; DRM, doramectin; EPM, eprinomectin; IVM, ivermectin; SEL, selamectin; MOX, moxidectin. For all strains and drugs evaluated, a minimum of three biological replicates were performed, each in technical duplicates.

^b^ Avermectin MICs were determined in the presence and absence of EDTA at the indicated concentrations.

^c^ MIC not determined as growth of the *E. coli* BW25113 Δ*bamB*Δ*tolC* strain was inhibited by the presence of 10 mM EDTA.

### EMB is bactericidal and bacteriolytic and causes severe cell envelope damage

**Fig 1:**
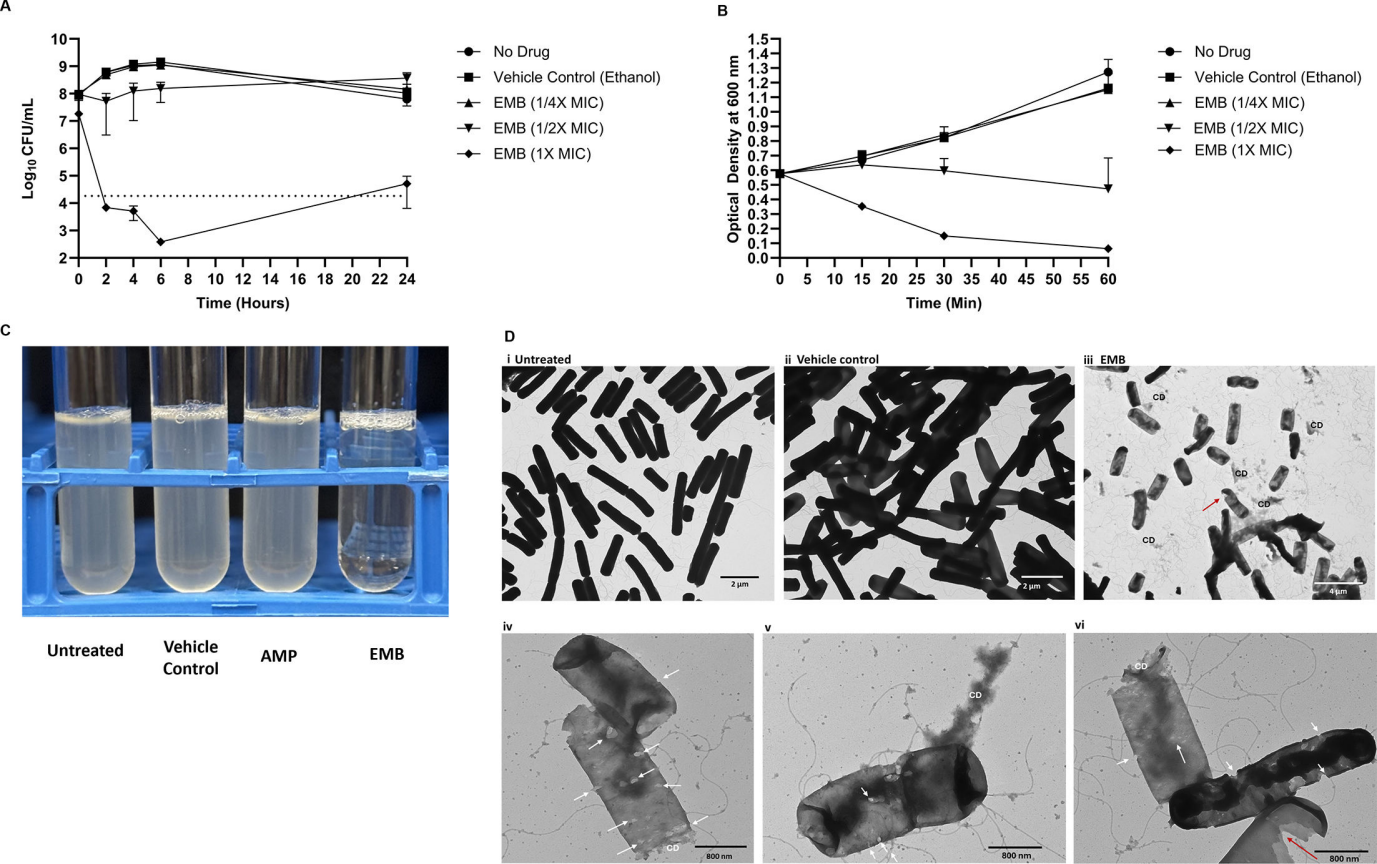
EMB is bactericidal and causes cell lysis of *B. subtilis* 1A1 cells. (**A**) Time-kill kinetics of EMB against *B. subtilis* were performed as outlined by CLSI (42). The dotted black line demarcates a 3-log reduction in CFU/mL relative to the initial (t = 0) inoculum of the 1× MIC of EMB-treated cells, and values below this line indicate bactericidal killing (i.e., 99.9% killing). (**B**) Cell lysis was monitored by measuring the optical density at 600 nm every 20 min following the addition of EMB at the indicated concentrations to exponential phase *B. subtilis* cells. For both (A) and (B), EMB concentrations are indicated by different symbols. Ethanol, which was used to solubilize EMB, was included as a vehicle control (at a similar concentration as used in 1× MIC EMB-exposed cells). (**C**) Treatment of *B. subtilis* with 1× MIC of EMB for 1 h resulted in lysis. Treatment of *B. subtilis* with 1× MIC of ampicillin (AMP; 0.125 µg/mL) did not visually impact cell turbidity. The figure is representative of 3 independent experiments. (**D**) TEM images of logarithmic *B. subtilis* cells treated, or not treated, with EMB for 30 min. (i) Untreated control; (ii) vehicle control (ethanol was added at the same concentration as used in the 1× MIC of EMB-treated cells); (iii) 1× MIC of EMB (32 µg/mL)-treated cells; and (iv) close-up TEM images of *B. subtilis* cells treated with 1× MIC of EMB. Red arrows indicate polar end separation, and white arrows indicate lesions in the cell envelope. CD, cell debris.

To elucidate whether EMB is bactericidal or bacteriostatic, a time-kill assay was performed using various concentrations of EMB against *B. subtilis*. EMB at 1× MIC exhibited bactericidal activity (Fig. 1A). We also monitored the turbidity of exponential phase cells treated with different concentrations of EMB at 0, 15, 30, and 60 min post-treatment (Fig. 1B). EMB at 1× MIC produced a rapid reduction in turbidity when compared with the untreated or vehicle-control (ethanol) treated cells, indicating cell lysis (Fig. 1B and C). Exposure of *B. subtilis* cells to 1/2× MIC of EMB resulted in a gradual decline in turbidity (Fig. 1B). The turbidity of the untreated, vehicle control-treated, and 1/4× MIC-treated cells increased similarly over time (Fig. 1B). TEM images of *B. subtilis* cells treated with 1× MIC of EMB revealed holes in the cell envelope, presence or expulsion of cell debris, detachment of cell ends, and lysis (Fig. 1D).

### EMB inhibits peptidoglycan synthesis

**Fig 2:**
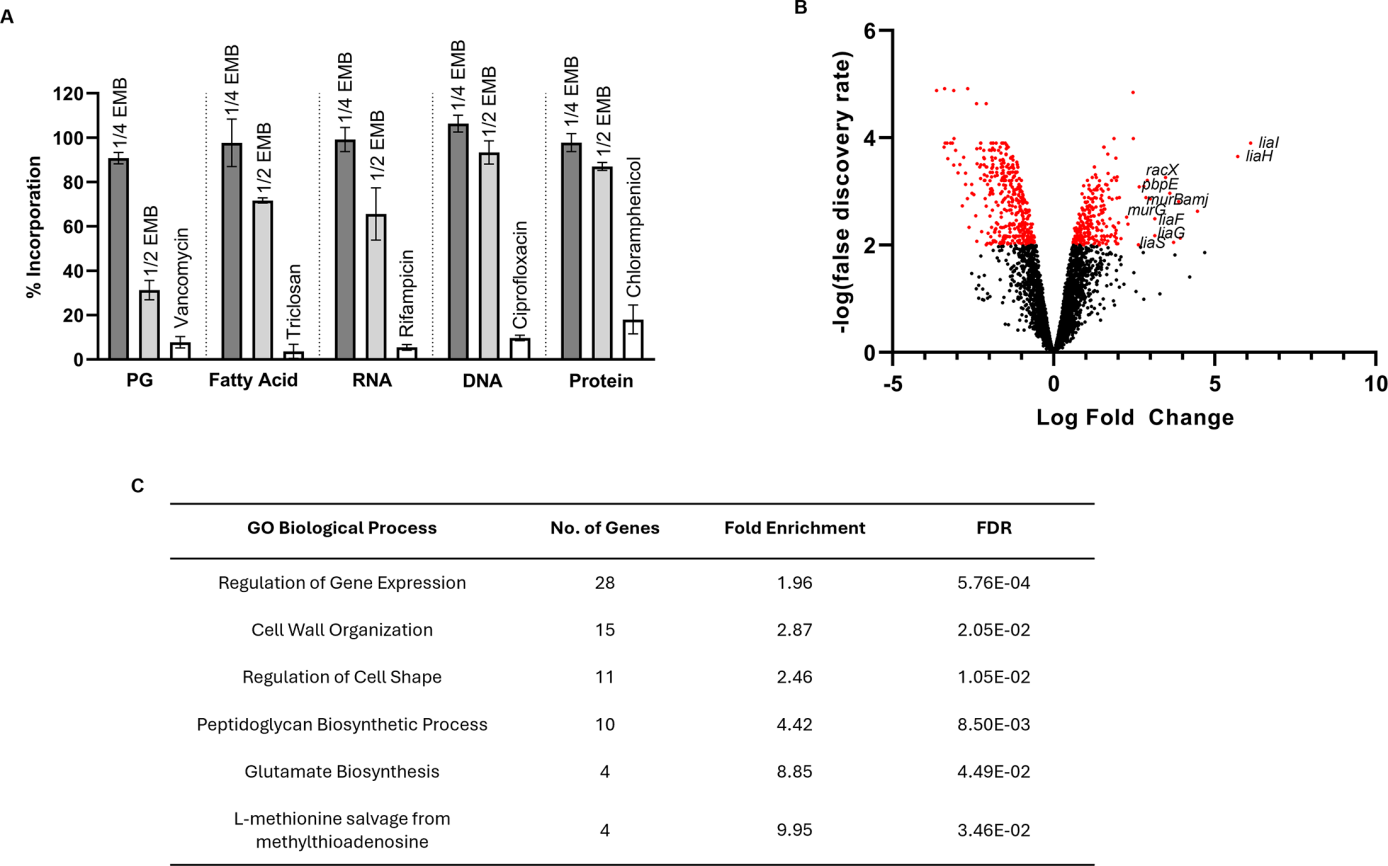
EMB interferes with PG synthesis and triggers a cell envelope stress response in *B. subtilis*. (**A**) Effect of EMB on macromolecular biosynthetic pathways in *B. subtilis*. Incorporation of [5,6-^3^H]-uridine (RNA), [3,4,5,-^3^H]-leucine (protein), [methyl-^3^H]-thymidine (DNA), [6-^3^H]-*N*-acetyl-D-glucosamine (PG, peptidoglycan), [1-^14^C]-acetic acid (fatty acid) was determined in *B. subtilis* treated with 1/4× (dark gray) or 1/2× MIC (light gray) of EMB. Vancomycin (4× MIC, 2 µg/mL), triclosan (4× MIC, 32 µg/mL), rifampicin (4× MIC, 0.5 µg/mL), ciprofloxacin (8× MIC, 1 µg/mL), and chloramphenicol [4× MIC, 16 µg/mL)] were used as positive controls (white bars). Data are averages ± SEM of at least four independent experiments. (**B**) Volcano plot represents RNA-seq analysis of exponential-phase WT *B. subtilis* after 30 min exposure to 8 µg/mL of EMB (1/4× MIC). The results are from four independent experiments. Significantly differentially expressed genes were fitted for significance with an FDR < 0.01 (i.e., -logFDR > 2) and are colored in red. Summary of all significantly differentially expressed genes can be found in File S1. (**C**) Gene Ontology (GO) analysis of significantly upregulated genes. Enriched GO terms were analyzed by Fisher’s exact test, and the results were filtered for significance with an FDR of <0.05.

Since resistance mutations can often be mapped to the molecular target of an antibiotic (52), several different methods were employed to select for EMB-resistant mutants (see Selection of EMB-resistant mutants of the Materials and Methods), but all failed to produce resistant isolates. To determine a potential biosynthetic pathway that EMB may inhibit, the effect of EMB on the incorporation of radiolabeled precursors into DNA, RNA, protein, lipid, and peptidoglycan (PG) was examined. As 1× MIC caused significant lysis 20 min post-treatment, sublethal concentrations of EMB could only be assessed. EMB at 1/2× MIC strongly inhibited PG biosynthesis and had a modest effect on fatty acid and RNA biosynthesis (Fig. 2A).

### EMB triggers an envelope stress response

Next, we turned to RNA sequencing (RNA-seq) experiments to generate hypotheses for the molecular target of EMB in *B. subtilis*. To accomplish this, exponential phase *B. subtilis* cells were treated with 1/4× MIC of EMB or the equivalent amount of solvent, ethanol (vehicle control), for 30 min prior to collecting the cells and extracting total RNA for RNA-seq; 1/4× MIC of EMB was used, as higher concentrations of EMB caused significant cell lysis following 30 min of exposure (Fig. 1B). A total of 658 genes were significantly differentially expressed in EMB-treated *B. subtilis* cells compared with vehicle control-treated cells (Fig. 2B and File S1). Of these, the expression of 258 genes was upregulated, whereas 400 were downregulated. To better understand how EMB kills the cells, we focused our analyses on transcripts that were upregulated by this compound. The overrepresented GO terms for biological process of upregulated genes were related to cell wall organization, regulation of cell shape, PG biosynthetic processes, glutamate biosynthetic process, and L-methionine salvage from methylthioadenosine (Fig. 2C). Notably, EMB induced expression of genes belonging to two cell envelope stress-responsive systems: the Lia system (*liaI*, *liaH*, *liaG*, *liaF*, and *liaS*) (53) and SigM regulon (e.g., *murB*, *amJ*, *tagT*, *divIB*, *recU, spx*, *yqjL*, *ypbG, and sigM*) (Fig. 2B and File S1) (54, 55). The PG biosynthetic genes *pbpE-racX*, *pbpA*, *murG*, and *murC* were also significantly upregulated in response to EMB (Fig. 2B and File S1).

To validate the RNA-seq data set, *B. subtilis* cells were similarly treated with 1/4× the MIC of EMB, and expression of *liaH*, *liaF*, *liaR*, *murG,* and *amj* was assessed using qRT-PCR. In agreement with the RNA-seq data, EMB significantly induced expression of *liaH*, *liaF*, *murG,* and *amj,* and not *liaR* (File S1 and Fig. S5).

### Inactivation of EMB-induced genes in *B. subtilis* does not alter susceptibility to EMB

To assess the contribution of the Lia system and non-essential PG biosynthetic genes to conferring resistance to EMB, the impact of inactivating the *liaS*, *liaR*, *liaI*, *liaH, pbpE*, *pbpA*, *racX*, and *amj* genes on EMB susceptibility was determined. Inactivation of these genes did not alter the sensitivity of *B. subtilis* to EMB (Table S1). Constitutive activation of the Lia system via inactivation of the negative regulator, *liaF* (56), also did not alter EMB susceptibility (Table S1).

### Knockdown of essential PG biosynthetic genes confers increased sensitivity to EMB

**Fig 3:**
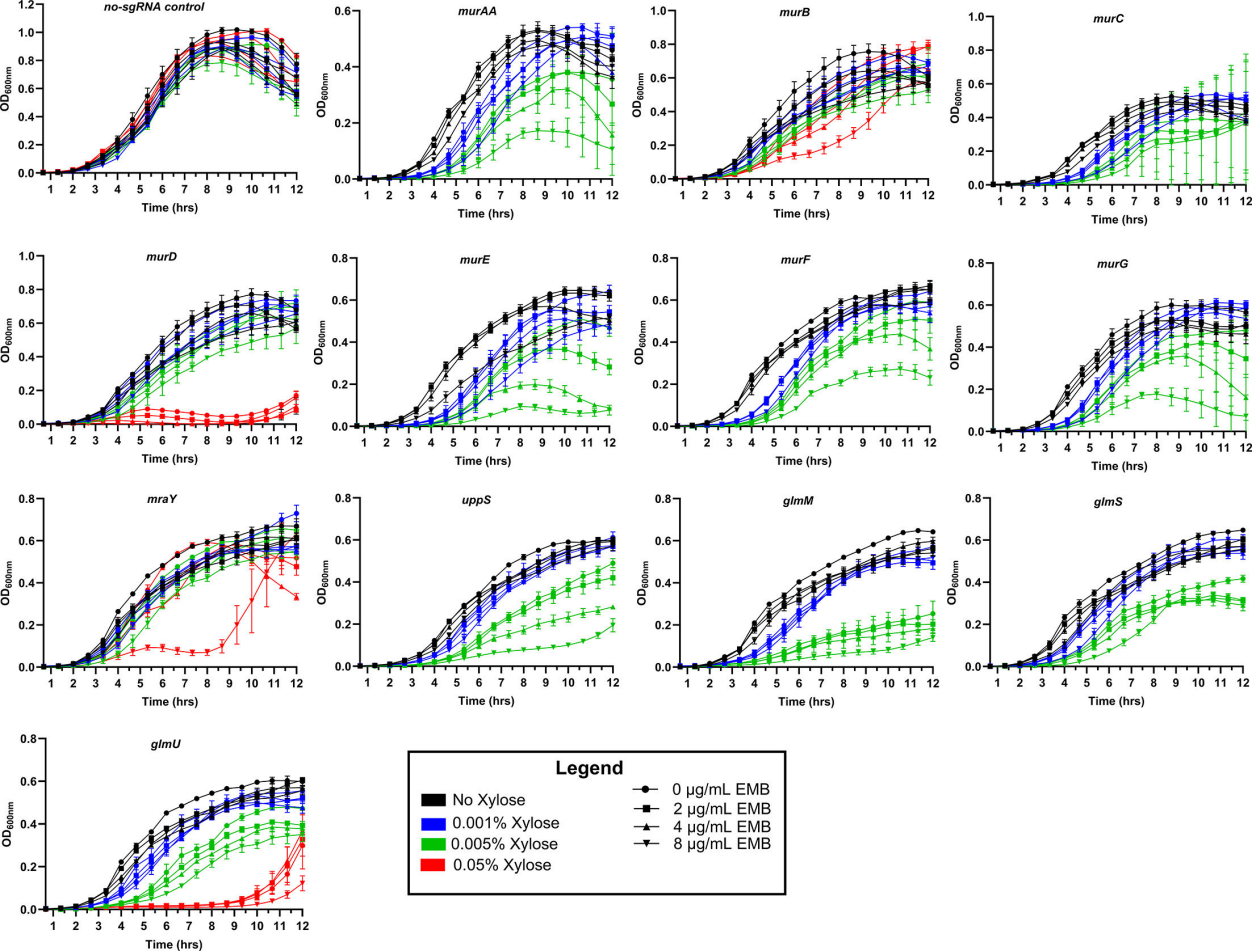
Knockdown of individual essential PG biosynthetic genes impairs the growth of *B. subtilis* cells exposed to sublethal concentrations of EMB. Data represent the means ± SEM of at least three biological replicates performed in technical triplicates. Optical densities at 600 nm (OD_600nm_) were baseline corrected by subtracting background absorbance at 600 nm of the uninoculated media. Black, blue, green, and red curves indicate growth in MHII broth without xylose, or with 0.001%, 0.005%, and 0.05% xylose, respectively. For improved visual clarity, symbols are shown at every other 20-min interval.

Given that EMB induced expression of PG biosynthetic genes, presumably to counteract cell wall damage, we examined the role of PG synthesis in conferring resistance to EMB. In bacteria, PG synthesis is a highly conserved, complex process that involves a series of essential stepwise enzymatic reactions that occur in the cytoplasm, cytoplasmic membrane, and extracellular space (Fig. S6) (57–59). Using previously constructed *B. subtilis* CRISPRi PG biosynthetic gene knockdowns (38), we assessed the impact of repressing the expression of several individual genes involved in PG synthesis on sensitizing *B. subtilis* to EMB using susceptibility testing (Table 5) and growth assays (Fig. 3). With the CRISPRi system, target gene expression levels are titratable and can be controlled by the addition of xylose to the media, from no xylose to a maximum of 1% (wt/vol) xylose resulting in basal (~3-fold repression) to full repression, respectively (38).

**TABLE 5:** Impact of CRISPRi-mediated repression of essential PG biosynthetic genes on EMB susceptibility in *B. subtilis*

	MIC (μg/mL)^a^																					
Drug	EMB				FOF		NIS		VAN		AMP		BAC		TM		DAP		CIP		TET	
Xylose (%)	0	0.001	0.005	0.01	0	0.01	0	0.01	0	0.01	0	0.01	0	0.01	0	0.01	0	0.01	0	0.01	0	0.01
Strain																						
WT	32	32	32	32	128	128	512	512	0.5	0.5	0.0625	0.0625	2048	2048	1	1	4	4	0.125	0.125	4	4
sgRNA^-^	32	32	32	32	128	128	512	512	0.5	0.25	0.0625	0.0625	1024	1024	1	1	4	4	0.125	0.125	4	4
*murAA*	32	32	**16** ^**b**^	-^d^	**<1**	**<1**	512	**256**	0.5	0.25	0.0625	**0.0312**	1024	1024	**0.5**	**0.25**	4	**2**	**0.0625**	**0.0625**	**2**	**1**
*murB*	32	32	32	32	128	**64**	512	512	0.5	0.25	0.0625	0.0625	1024	1024	1	**0.5**	4	**2**	**0.0625**	**0.0625**	4	4
*murC*	32	32	-^c^	-^e^	**64**	**<1**	512	**256**	0.5	0.25	0.0625	**0.0312**	1024	1024	**0.5**	**0.25**	4	**≤0.0312**	**0.0625**	**0.0625**	**2**	**1**
*murD*	32	32	**16**	**8/16** ^**f**^	128	**64**	512	512	0.5	0.25	0.0625	**0.0312**	1024	1024	1	**0.5**	4	**2**	**0.0625**	**0.0625**	4	4
*murE*	32	32	**8**	**4**	**64**	**16**	512	**256**	0.5	0.25	0.0625	**0.0312**	1024	1024	**0.5**	**0.5**	2/4^f^	**2**	**0.0625**	**0.0312**	**2**	**2**
*murF*	32	32	**16**	**8**	128	**32**	512	**256**	0.5	0.25	0.0625	**0.0312**	1024	1024	1	**0.5**	4	**2**	**0.0625**	**0.0625**	**2**	**2**
*murG*	32	32	**16**	**4**	128	**32**	512	**256**	0.5	0.25	0.0625	0.0625	1024	1024	1	**0.5**	4	**2**	**0.0625**	**0.0625**	**2**	**2**
*mraY*	32	32	32	32	128	**64**	512	512	0.5	0.25	0.0625	**0.0312**	1024	1024	1	**0.5**	4	**2**	**0.0625**	**0.0625**	4	4
*glmM*	32	32	**16**	**16**	**64**	**32**	512	512	0.5	0.25	0.0625	0.0625	1024	1024	**0.5**	**0.25**	4	**2**	**0.0625**	**0.0625**	4	**2**
*glmS*	32	32	**16**	**16**	256	256	512	512	0.5	0.25	0.0625	**0.0312**	1024	1024	**0.5**	**0.5**	4	**2**	**0.0625**	**0.0625**	4	**2**
*glmU*	32	32	**16**	**16**	256	**64**	512	**256**	0.5	0.25	0.125	0.0625	1024	1024	1	**0.5**	4	**2**	**0.0625**	**0.0625**	4	**2**
*uppS*	32	32	32	32	128	**32**	512	**256**	0.5	0.25	0.0625	**0.0312**	1024	1024	1	**0.5**	4	**2**	**0.0625**	**0.0625**	**2**	**2**

^a^ Bolded values indicate a decrease in MIC of the corresponding knockdown strain relative to the No-sgRNA control strain (sgRNA^-^). EMB, emamectin benzoate; FOF, fosfomycin; NIS, nisin; VAN, vancomycin; AMP, ampicillin; BAC, bacitracin-zinc; TM, tunicamycin; DAP, daptomycin. For all strains evaluated, a minimum of two biological replicates were performed, each in technical duplicates. WT, *B. subtilis* 1A1.

^b^ When cultured in MHII broth supplemented with 0.005% xylose, the *murAA* knockdown visibly grew at 0.125, 0.25, 0.5, 1 and 8 µg/mL but not at 2, 4 and ≥ 16 µg/mL of EMB for all biological and technical replicates. Therefore, the MIC was interpreted as 16 µg/mL.

^c^ When cultured in MHII broth supplemented with 0.01% xylose, growth of the *murAA* knockdown at concentrations between 0.125 and 4 µg/mL was sporadic, with skipped wells observed in both technical and biological replicates. This made interpretation of an MIC challenging. Therefore, an MIC is not reported for this knockdown under the conditions tested.

^d^ When cultured in MHII broth supplemented with 0.005% xylose, growth of the *murC* knockdown at concentrations between 0.125 and 4 µg/mL was sporadic, with multiple skipped wells observed in both technical and biological replicates. This made the interpretation of an MIC challenging. Therefore, an MIC is not reported for this knockdown under the conditions tested.

^e^ When cultured in MHII broth supplemented with 0.01% xylose, growth of the *murC* knockdown at concentrations between 0.125 and 1 µg/mL was sporadic, with skipped wells observed in both technical and biological replicates. This made the interpretation of an MIC challenging. Therefore, an MIC is not reported for this knockdown under the conditions tested.

^f^ The corresponding MIC among technical replicates performed in biological duplicates differed by 2-fold.

Basal repression or low-level repression (i.e., 0.001% xylose) of the PG biosynthetic genes did not sensitize *B. subtilis* to EMB (Table 5). Higher xylose concentrations (0.005% or 0.01%) decreased the EMB MICs of the *murAA*, *murD-G*, *glmM*, *glmS*, and *glmU* knockdowns compared with the no-sgRNA control and WT *B. subtilis* strains (Table 5). EMB MICs for *murC* (0.005% and 0.01% xylose) and *murAA* (0.01% xylose) could not accurately be determined due to inconsistent, multiple-skipped well phenotypes among technical and biological replicates, despite growth in xylose alone. Finally, knockdown of *mraY* and *murB* did not alter the sensitivity of *B. subtilis* to EMB under all conditions examined.

The growth of the knockdown strains exposed to sublethal concentrations of EMB under different levels of induction was also monitored (Fig. 3). We confirmed that sublethal concentrations of EMB did not adversely impact the growth of the no-sgRNA control strain under all conditions tested. Overall, under high induction conditions (0.005% xylose), the growth of all knockdowns, except for *murC*, *murB,* and *mraY*, was negatively affected by sublethal concentrations of EMB relative to untreated cells. Growth of the *murC* knockdown was highly variable in the presence of 0.005% xylose alone and is consistent with our inability to accurately determine an EMB MIC for this strain (Fig. 3 and Table 5). However, under low induction (0.001% xylose), the growth of the *murC* knockdown was affected by 8 µg/mL of EMB. The simplest explanation for the *mraY* and *murB* knockdowns remaining unaffected by sublethal concentration of EMB is that xylose concentrations were not sufficient to reduce *murB* and *mraY* expression levels to produce an observable effect. In agreement with this, sublethal concentrations of EMB negatively impacted the growth of the *murB* and *mraY* knockdowns when cultured in the presence of a higher concentration of xylose (0.1%) (Fig. 3). Altogether, when accounting for both susceptibility testing results and growth curve analyses, interfering with several individual steps of PG synthesis sensitizes *B. subtilis* to EMB.

To assess whether the pattern of sensitivity among knockdowns to EMB resembles that of a known PG synthesis inhibitor, and therefore have the same target, the susceptibility of the knockdowns to antibiotics that target the cytoplasmic (fosfomycin, tunicamycin), membrane-bound (nisin, bacitracin-zinc, vancomycin), and extracellular (ampicillin) steps of PG synthesis in the absence or presence of 0.01% xylose was determined (Table 5). Several knockdowns demonstrated an increase in susceptibility to fosfomycin, with *murAA* [encoding for fosfomycin’s target (60)] demonstrating the greatest susceptibility to this agent even in the absence of xylose. Tunicamycin MICs were either 2-fold or 4-fold lower for all knockdowns, including *mraY*, which encodes for the target of tunicamycin (61), relative to the no-sgRNA control when cultured with xylose. In the presence of xylose, the MICs for nisin, daptomycin, and ampicillin decreased 2-fold for several knockdown strains. Vancomycin and bacitracin-zinc MICs for all knockdowns were indistinguishable from the no sgRNA-control under all conditions tested. The DNA and protein synthesis inhibitors, ciprofloxacin and tetracycline, respectively, were also included as non-cell wall-targeting antibiotic controls. However, both drugs have previously been shown to have secondary effects on PG synthesis (62–64). All knockdowns demonstrated a decrease in ciprofloxacin MICs relative to the no-sgRNA control under all conditions tested. Nine knockdowns demonstrated a 2-fold to 4-fold increase in tetracycline susceptibility relative to the no-sgRNA control strain when cultured with xylose. These observations are consistent with ciprofloxacin and tetracycline affecting cell wall synthesis (62–64). Overall, when compared with the cell wall synthesis inhibitors with known modes of action, the pattern of sensitivity among knockdowns appears to be unique to EMB, especially for the *murAA* and *murC* knockdowns. Still*,* it is unclear whether the genetic repression of the individual PG biosynthetic genes themselves or secondary effects related to their repression sensitizes *B. subtilis* to EMB.

### PG synthesis inhibitors produce synergy with EMB

Examination of pairwise antibiotic interactions has previously been used to gain insight into antibiotic’s mechanism of action (65). Using checkerboard assays, we examined the interaction of EMB with twelve different antibiotics for synergistic activity against *B. subtilis* by calculating the fractional inhibitory concentration index. An FICI value of ≤0.5 or >0.5–4 was reported as synergistic or nonsynergistic, respectively (66). Of the antibiotics examined, seven target different stages of cell wall biosynthesis (fosfomycin, tunicamycin, vancomycin, bacitracin-zinc, nisin, daptomycin, and ampicillin), three target protein synthesis (erythromycin, tetracycline, and chloramphenicol), and one targets either RNA synthesis (rifampicin) or DNA synthesis (ciprofloxacin). As seen in Table 6, combinations of EMB with fosfomycin, tunicamycin, nisin, and daptomycin produced synergism against *B. subtilis*. No synergy was observed with EMB in combination with all other antibiotics examined.

**TABLE 6:** Fractional inhibitory concentrations of EMB in combination with select antibiotics against *B. subtilis*

Agent	MIC (μg/mL)^a^		FIC^b^	FICI^c^	Interpretation
	Alone	Combination			
EMB	32	16	0.5	1	Nonsynergistic
Vancomycin	0.5	0.25	0.5		
EMB	32	8	0.25	0.375	Synergistic
Nisin	512	64	0.125		
EMB	32	16	0.5	0.563	Nonsynergistic
Bacitracin-zinc	2048	128	0.0625		
EMB	32	0.5	0.0156	0.266	Synergistic
Fosfomycin	256	64	0.25		
EMB	32	16	0.5	0.625	Nonsynergistic
Ampicillin	0.125	0.0156	0.125		
EMB	32	8	0.25	0.5	Synergistic
Daptomycin	4	1	0.25		
EMB	32	8	0.25	0.5	Synergistic
Tunicamycin	0.5	0.125	0.25		
EMB	32	32	1	2	Nonsynergistic
Erythromycin	0.125	0.125	1		
EMB	32	32	1	2	Nonsynergistic
Chloramphenicol	4	4	1		
EMB	32	16	0.5	1	Nonsynergistic
Tetracycline	4	2	0.5		
EMB	32	32	1	2	Nonsynergistic
Ciprofloxacin	0.125	0.125	1		
EMB	32	16	0.5	0.625	Nonsynergistic
Rifampicin	0.125	0.0156	0.125		

^a^ EMB, emamectin benzoate. For all antibiotic combinations tested against *B. subtilis*, three biological replicates were performed.

^b^ Fractional Inhibitory Concentration (FIC) is either MIC of drug A when combined with drug B divided by the MIC of drug A alone (FIC A) or the MIC of drug B when combined with drug A divided by the MIC of drug B alone (FIC B).

^c^ FICI (FIC A + FIC B) value of ≤0.5 was defined as synergistic; FICI ranging from >0.5-4 was defined as nonsynergistic.

### Phosphatidylglycerol antagonizes the action of EMB against *B. subtilis*

Since recovering EMB-resistant mutants was difficult, a common feature for antibiotics that directly interact with membrane phospholipids or cell wall precursors (67–71), we examined whether several purified cell envelope components could antagonize the action of EMB and thus serve as potential target molecules. Preincubation of EMB with phosphatidylglycerol, a main lipid component of the bacterial membrane, antagonized the antibacterial activity of EMB (i.e., restored growth of *B. subtilis* in the presence of 1× MIC of EMB), whereas purified cell wall components did not (Table 7).

**TABLE 7:** Growth of *B. subtilis* in the presence of EMB and cell envelope components

Antagonist	Antagonist:EMB^a^					
	0:1	0.5:1	1:1	2:1	4:1	10:1
*N*-acetylglucosamine	NG	NG	NG	NG	NG	NG
*N*-acetylmuramic acid	NG	NG	NG	NG	NG	NG
Ala-D-γ-Glu-Lys-D-Ala-D-Ala	NG	NG	NG	NG	NG	NG
Peptidoglycan from *Bacillus subtilis*	NG	NG	NG	NG	NG	NG
Undecaprenyl-monophosphate (UP)	NG	NG	NG	ND	ND	ND
Undecaprenyl-diphosphate (UPP)	NG	NG	NG	NG	NG	ND
Phosphatidylglycerol	NG	NG	G	G	G	ND

^a^ *B. subtilis* was incubated with EMB at 1x MIC (32 µg/mL) in MHII broth in the presence or absence of putative purified antagonists, which were added at the specified weight/volume (μg/mL) ratios relative to the concentration of EMB. NG, no growth/no antagonism; G, growth/antagonism; ND, not determined. At least three biological replicates were performed in technical duplicates.

## DISCUSSION

In contrast to previous findings (34), we demonstrated that avermectins and MOX exhibit anti-gram-positive activity, albeit with varying degrees of potency and selectivity (Table 3). Previously, Lim et al. reported that SEL, DRM, IVM, and MOX had excellent activity against clinical strains of *M. tuberculosis* but lacked activity against gram-positive bacteria, including *Streptomyces lividans*, *Rhodococcus jostii* RHA1, *Kocuria rhizophila,* and *S. aureus* ATCC 25923 (34). With the exception of *S. aureus* ATCC 25923, none of the other previously examined gram-positive species were included in the present study. We found that SEL exhibited bacteriostatic activity against *S. aureus* ATCC 25923 and inhibited the growth of *S. epidermidis* (Fig. S4 and Table 3). In agreement with this, SEL was recently shown to be active against clinical strains of *S. aureus* (37). The conflicting findings reported by Lim et al. may be due to differences in MIC endpoint determination methods. Lim et al. used the bacterial growth indicator MTT [3-(4,5-dimethylthiazol-2-yl)−2,5-diphenyl tetrazolium bromide] to determine the concentration of drug that inhibited growth by 90% (34), whereas visual inspection and turbidity were used in the present study and by Folliero et al. (37), respectively, to interpret MICs. The MTT assay is based on the principle that enzymes present in metabolically active cells reduce the water-soluble, yellowish tetrazolium salt to water-insoluble, purple formazan crystals (72). Formazan production is directly proportional to the number of viable cells and can be quantified spectrophotometrically (72). Several factors, including the culture medium (72), solvent used to solubilize formazan (73), and the test compounds themselves (74, 75) may interfere with the MTT assay, leading to false estimations of cell viability (76). It is recommended that MTT assay results be interpreted with caution and validated with other non-metabolic assays (72, 75). One possiblility, then, for the previously reported SEL MIC of >256 µg/mL for *S. aureus* is that the low solubility, and thus reduced bioavailability, of SEL at concentrations ranging from 32 to 256 µg/mL allowed *S. aureus* to grow, which was then detected by the MTT assay (34).

Of the avermectins examined, EPM is structurally the most similar to EMB. EPM contains an acetamido group at the 4” position of the terminal saccharide group, whereas EMB contains a methylamino group at this position (Fig. S2). Therefore, the methylamino group of EMB appears to be important for its anti-gram-positive activity and potentially its interaction with phosphatidylglycerol. Indeed, chemical modification of the 4” position of the terminal saccharide has previously been shown to alter the biological activity of avermectins (77–79). The structure of SEL is unique compared to other avermectins, as it contains a monosaccharide unit (instead of a disaccharide unit) and an oxime group (instead of a hydroxyl group) at positions C13 and C5, respectively (Fig. S2). Although it is unclear how these structural differences relate to variations in antibiotic activity, chemical modifications at positions C4”, C13, and C5 may prove useful for the development of novel semi-synthetic antibacterial avermectins.

**Fig 4:**
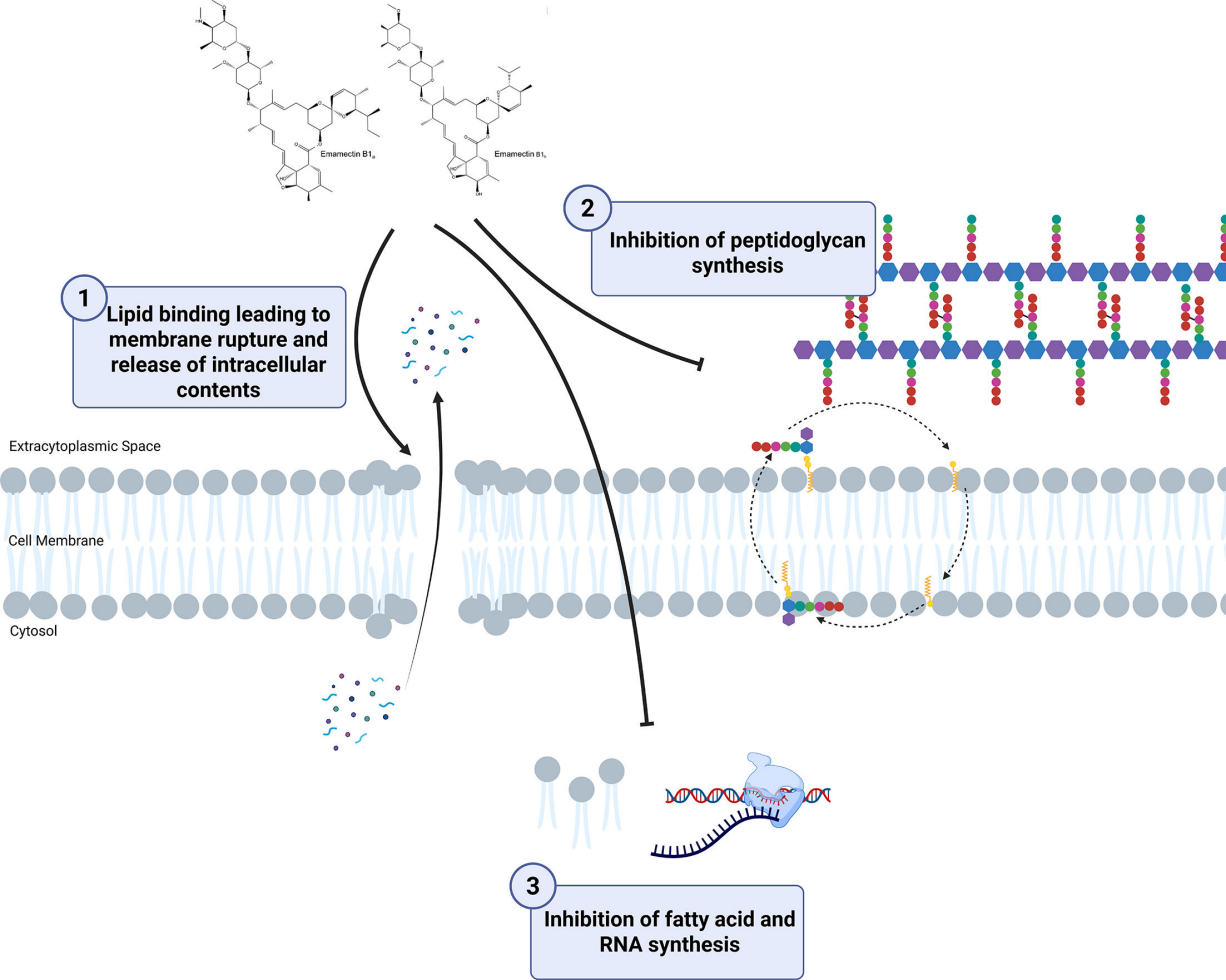
Proposed mode of action of EMB against gram-positive bacteria. Positively charged EMB directly interacts with negatively charged phosphatidylglycerol and potentially other anionic phospholipids (e.g., cardiolipin) in the cytoplasmic membrane, resulting in membrane rupture and release of intracellular contents. Inhibition of PG synthesis may be direct and/or indirect. Direct effects on PG synthesis may involve EMB’s interaction with cell wall precursors or components of the PG biosynthetic machinery. Indirect effects on PG synthesis may involve EMB-mediated membrane disruption, which triggers the delocalization of membrane-bound PG biosynthetic machinery. Finally, EMB partially inhibits fatty acid and RNA synthesis. Created in BioRender. Fruci, M. (2025) https://BioRender.com/d5hxaul.

We provide several lines of evidence indicating that the cell envelope is the primary target of EMB, interfering with both membrane and cell wall functions. Given that EMB is positively charged in solution (80), we postulate that EMB binds to anionic phospholipids (e.g., phosphatidylglycerol) in the cytoplasmic membrane, resulting in the formation of holes and the leakage of cytoplasmic contents (Fig. 4). EMB may then inhibit PG biosynthesis either directly or indirectly. Direct effects on PG synthesis may involve EMB’s interaction with either PG precursor molecules (e.g., lipid II, which was not examined in this study) or essential PG biosynthetic enzymes. Indirect effects may involve EMB-mediated alterations to the overall stability of the membrane, triggering the delocalization of membrane-bound PG biosynthetic machinery. The exact mechanism(s) by which EMB inhibits PG synthesis requires further investigation. EMB’s likely interaction with the cytoplasmic membrane would also explain its spectrum of activity against gram-positive bacteria and outer membrane-permeabilized *E. coli* cells, where the cytoplasmic membrane, which also contains phosphatidylglycerol (81), is exposed. We also cannot disregard EMB’s modest effects on fatty acid and RNA biosynthesis in contributing to its mechanism of action. Additionally, several studies have suggested that reactive oxygen species generated by bactericidal antibiotics may contribute to antibiotic killing (82). Altogether, a more detailed investigation of the mechanism of action of EMB is warranted.

The failure to recover resistant mutants is a typical feature shared among membrane- (67) and PG precursor-targeting antibiotics (68–71), but not of protein-targeting antibiotics (52). This is consistent with the potential target of EMB being phosphatidylglycerol. In a recent study by Ezquerra-Aznárez et al., exposure of a wild-type strain of *Mycobacterium smegmatis* to SEL failed to yield SEL-resistant mutants (83). However, exposure of a *M. smegmatis* Δ*nucS* mutator strain produced SEL-intermediate resistant mutants harboring nonsense mutations in genes involved in cell envelope lipid biogenesis, *mmpL11* and *mps1* (83). Introduction of these mutations into a *M. smegmatis* Δ*nucS* background strain recapitulated the resistance phenotype (83). Although high-level SEL-resistant mutants were derived from the intermediate-SEL-resistant mutants, mutations responsible for conferring high-level SEL resistance were not identified (83). Notably, SEL-resistant mutants demonstrated changes in the composition of outer membrane glycopeptidolipids (83). Such changes in the cell envelope composition of SEL-resistant mutants were hypothesized to reduce outer membrane permeability and thus uptake of SEL (83). Although the target of SEL remains elusive, increasing cell wall permeability in the WT *M. smegmatis* strain by deleting the arabinosyltransferase B encoding gene, *embB*, did not significantly affect SEL susceptibility (83). This suggests that SEL’s target is not cytoplasmic, and like EMB, may reside in the cell envelope (83). Nevertheless, we suspect that exposing a mutator strain of *B. subtilis* to EMB may favor the recovery of presumably rare, EMB-resistant bacterial mutants, potentially with modified lipid membrane content or charge. This should be challenged with future experiments.

Consistent with EMB causing cell envelope damage, both the cell envelope stress-sensing Lia and SigM systems were activated in *B. subtilis* cells upon exposure to EMB (Fig. 2B and File S1). The Lia system is comprised of a histidine sensor kinase, LiaS, a response regulator, LiaR, and a membrane-anchored negative regulator, LiaF (84, 85). The system is encoded by the last three genes of the hexacistronic locus, *liaIH-liaGFSR*, which is expressed as two transcriptional units (*liaIH* and *liaIHGFSR*) (84, 85). The Lia system is activated by PG precursor-binding inhibitors (e.g., vancomycin, bacitracin, daptomycin, nisin, and ramoplanin) (53, 84, 86–88) and by membrane-disrupting agents (e.g., diphenyl ether, n-hexane, cyclooctane, and benzyldimethylhexadecylammonium chloride (84). Upon activation, LiaR promotes expression of the *liaIH* operon, the only relevant target of the LiaFSR system in *B. subtilis* (53, 84). LiaI, a small-membrane protein, has been shown to recruit and co-localize with LiaH at static spots in the cytoplasmic membrane where they counteract antibiotic-induced cell envelope damage, presumably by stabilizing the membrane (53, 85, 89). The Lia system does not confer resistance against many of its inducers, except for daptomycin and enduracidin (53, 86). This is consistent with the observation that EMB activates the Lia system, but the Lia system does not confer EMB resistance (Table S1).

In *B. subtilis*, SigM is activated by ethanol, heat, acid, redox cycling agents (e.g., paraquat), and virtually all PG synthesis inhibitors (55, 90) and controls expression of genes involved in the synthesis of PG precursors, PG assembly or modification, and cell division (91, 92). Recently, SigM and its membrane-anchored anti-sigma factor, YhdL-YhdK, were shown to respond to antibiotic-mediated reduction in undecaprenyl phosphate (UP) levels (55). When UP levels are reduced, SigM is released from its membrane-anchored anti-sigma factor complex and activates the expression of genes that increase PG synthesis, promote UP recycling, and liberate UP from participating in the translocation of nonessential surface polymer pathways (55). In this context, EMB-mediated activation of the SigM response may be due to its potential effect on UP levels. Depletion of phosphatidylglycerol has also been shown to activate the SigM response in *B. subtilis*, an indication that SigM responds to alterations in membrane lipid composition (93). In this vein, EMB’s interaction and potential sequestration of phosphatidylglycerol may contribute to the activation of SigM. Nevertheless, EMB-mediated activation of the SigM response is consistent with this agent damaging the cell envelope.

EMB also induced expression of the two-gene operon *pbpE-racX* and *pbpA*, presumably to counteract cell wall damage (Fig. 2B and File S1). Individual knockouts of these genes in *B. subtilis* did not alter the susceptibility of *B. subtilis* to EMB, likely due to compensatory mechanisms (Table S1). The gene *pbpE* encodes for penicillin-binding protein 4^*^ (PBP4^*^) endopeptidase involved in PG recycling, and the gene *racX* encodes for an amino acid racemase that produces non-canonical D-amino acids, but its physiological role is still unknown (94, 95). Expression of *pbpE-racX* is inducible by vancomycin (88), detergents, and membrane-targeting cationic antimicrobial peptides (96). Despite this operon being regulated by the extracytoplasmic function sigma factor, SigW, which is implicated in maintaining cell envelope integrity (97), EMB did not significantly induce expression of *sigW* (File S1). The gene *pbpA* encodes for PBP2a and is implicated in elongation-specific PG synthesis in *B. subtilis* (98). Another penicillin-binding protein, PbpH, is functionally analogous to PBP2a, and therefore it is not surprising that inactivation of *pbpA* does not alter EMB susceptibility of *B. subtilis* (99). Although EMB induced the expression of the lipid II flippase encoding gene*, amj* (Fig. 2B and File S1), inactivation of *amj* did not alter the susceptibility of *B. subtilis* to EMB (Table S1), likely due to the presence of its functional equivalent, MurJ (100). Taken together, increased expression of these PG biosynthetic enzymes with redundant functions likely allows cells to continue PG synthesis and maintain cell wall integrity upon exposure to sublethal concentrations of EMB.

The PG synthesis inhibitors fosfomycin, tunicamycin, nisin, and daptomycin produced synergism with EMB against *B. subtilis* (Table 6). Synergistic interactions can be explained by two drugs acting on different steps of the same essential pathway (e.g., sulfamethoxazole and trimethoprim) (101). Therefore, EMB may work synergistically with PG synthesis inhibitors by targeting different steps in PG synthesis. Synergy may also be explained by one drug enhancing the intracellular uptake of another drug (102, 103). Of the antibiotics that produced synergy with EMB, fosfomycin was the only antibiotic with a cytoplasmic target (60). However, EMB did not synergize with other antibiotics with cytoplasmic targets, suggesting that EMB may also work in concert with fosfomycin by perturbing cell wall synthesis. Despite the expectation that vancomycin, ampicillin, and bacitracin-zinc would also synergize with EMB, they did not. Several studies have found a discordance between different *in vitro* synergy testing methods, thus influencing whether a drug combination is deemed synergistic (104, 105). Therefore, comparing different synergy testing methods may yield different results, especially for ampicillin and bacitracin-zinc, whose FICI values bordered the synergism threshold. It is worth noting that nonsynergistic interactions do not necessarily inform the mechanism of action. For example, owing to the greater number of PBPs with varying affinities for different β-lactams found in gram-negative bacteria, synergistic interactions among β-lactams are common in gram-negative species but rare in gram-positive species (106). As such, antibiotics targeting the same biosynthetic pathway may not produce synergism in every instance; therefore, a lack of interaction should be interpreted with caution (106).

As EMB is toxic in humans (107), it is unlikely to be repurposed as an antibiotic for clinical use. However, due to the lack of new antibiotics and the emergence of multidrug-resistant bacterial pathogens, EMB may serve as a scaffold for the development of new, non-toxic, semi-synthetic antibacterial avermectins. Future studies examining the ability of avermectins and milbemycins, especially those already approved for veterinary and human medicine, to kill drug-resistant gram-positive pathogens, either individually or in combination with clinically important antibiotics, are required. Finally, and concerningly, given the intensive use of avermectins in agriculture, aquaculture, and human and veterinary medicine, consideration should be given to examining the off-target effects of these agents on the composition and function of various bacterial communities.

## AFTER EPUB

[This article was published on 2 September 2025 with an error in the supplemental file. The error was corrected in the current version, posted on 10 September 2025.]

## SUPPLEMENTAL MATERIAL

File S1 spectrum.00274-25-s0001.xlsx RNA-seq analysis of significantly differentially regulated genes of *B. subtilis* in response to emamectin benzoate.

Figures S1 to S6, and Table S1 spectrum.00274-25-s0002.docx File contains chemical structures of avermectins and moxidectin, optical densities of 96-well microtiter susceptibility testing plates, time-kill assay of selamectin against *S. aureus*, qPCR data, and schematic of cell wall synthesis.

ASM does not own the copyrights to Supplemental Material that may be linked to, or accessed through, an article. The authors have granted ASM a non-exclusive, world-wide license to publish the Supplemental Material files. Please contact the corresponding author directly for reuse.
