# Supplementary material for: The avermectin, emamectin benzoate, kills gram-positive bacteria and targets the cell envelope of Bacillus subtilis
Source: Microbiol Spectr. 2025 Sep 2;13(10):e00274-25. doi: 10.1128/spectrum.00274-25 (PMC12502619; doi:10.1128/spectrum.00274-25)
Supplement: Figures S1 to S6, and Table S1 — File contains chemical structures of avermectins and moxidectin, optical densities of 96-well microtiter susceptibility testing plates, time-kill assay of selamectin against S. aureus, qPCR data, and schematic of cell wall synthesis. [file spectrum.00274-25-s0002.docx]

**Supplemental Figures and Tables**

**
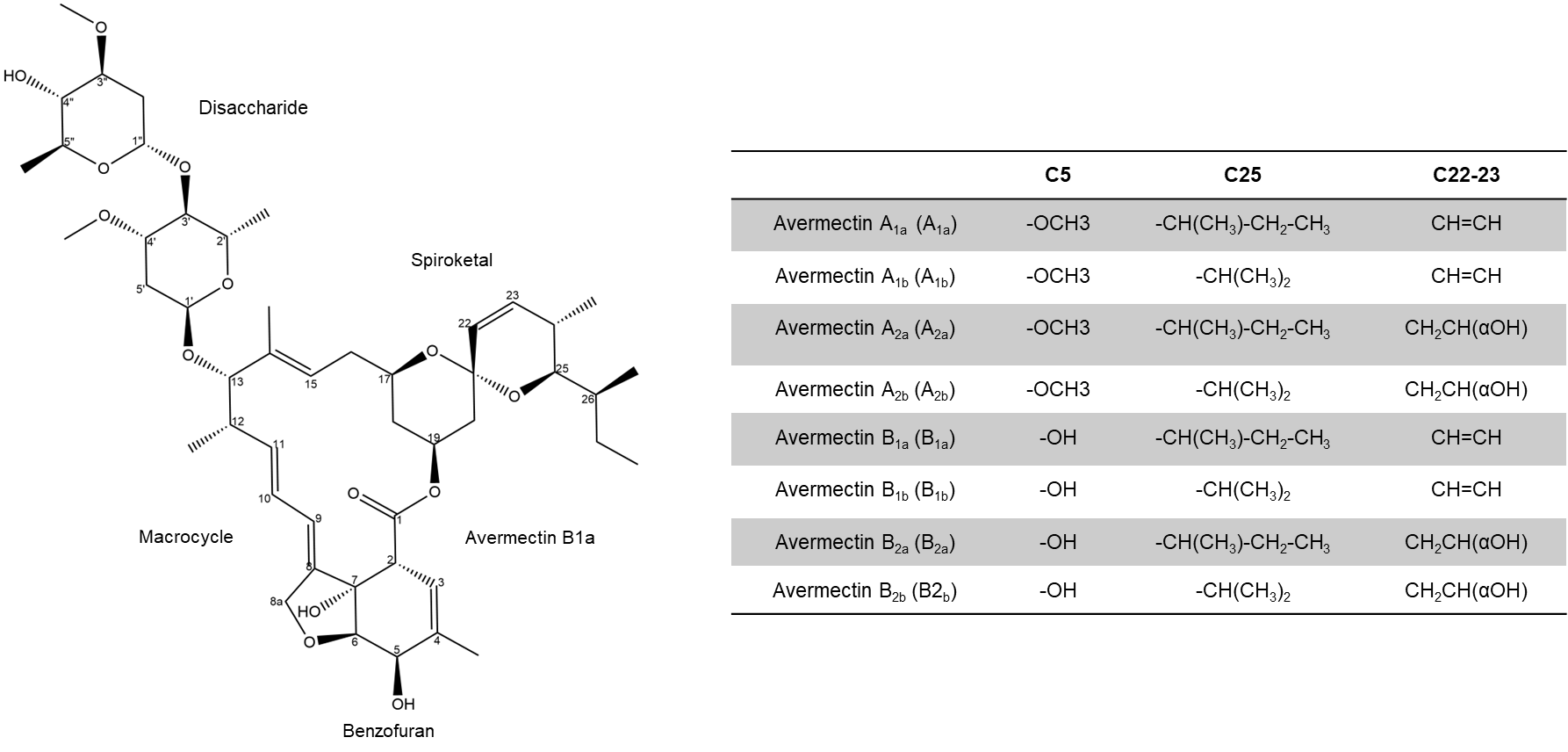
**

**Fig S1.** Chemical structure of avermectin B1a and different groups at positions C5, C25 and C22-23 of the eight different avermectin compounds. The ‘A’- and ‘B’-series compounds contain a methoxy (-OCH3) or hydroxy (-OH) group at position C5, respectively. The ‘1’- and ‘2’- compounds have a double bond between C22-C23 (CH=CH), or a single bond between C22-C23 and a hydroxyl group (CH_2_CH(αOH)) at position C23. The ‘a’ and ‘b’ designations indicate a secbutyl [-CH(CH_3_)-CH_2_-CH_3_] or isopropyl [-CH(CH_3_)_2_] group at position C25, respectively. Chemical structure was drawn with ChemDraw v.20.0 using the PubChem CID, 6434889.


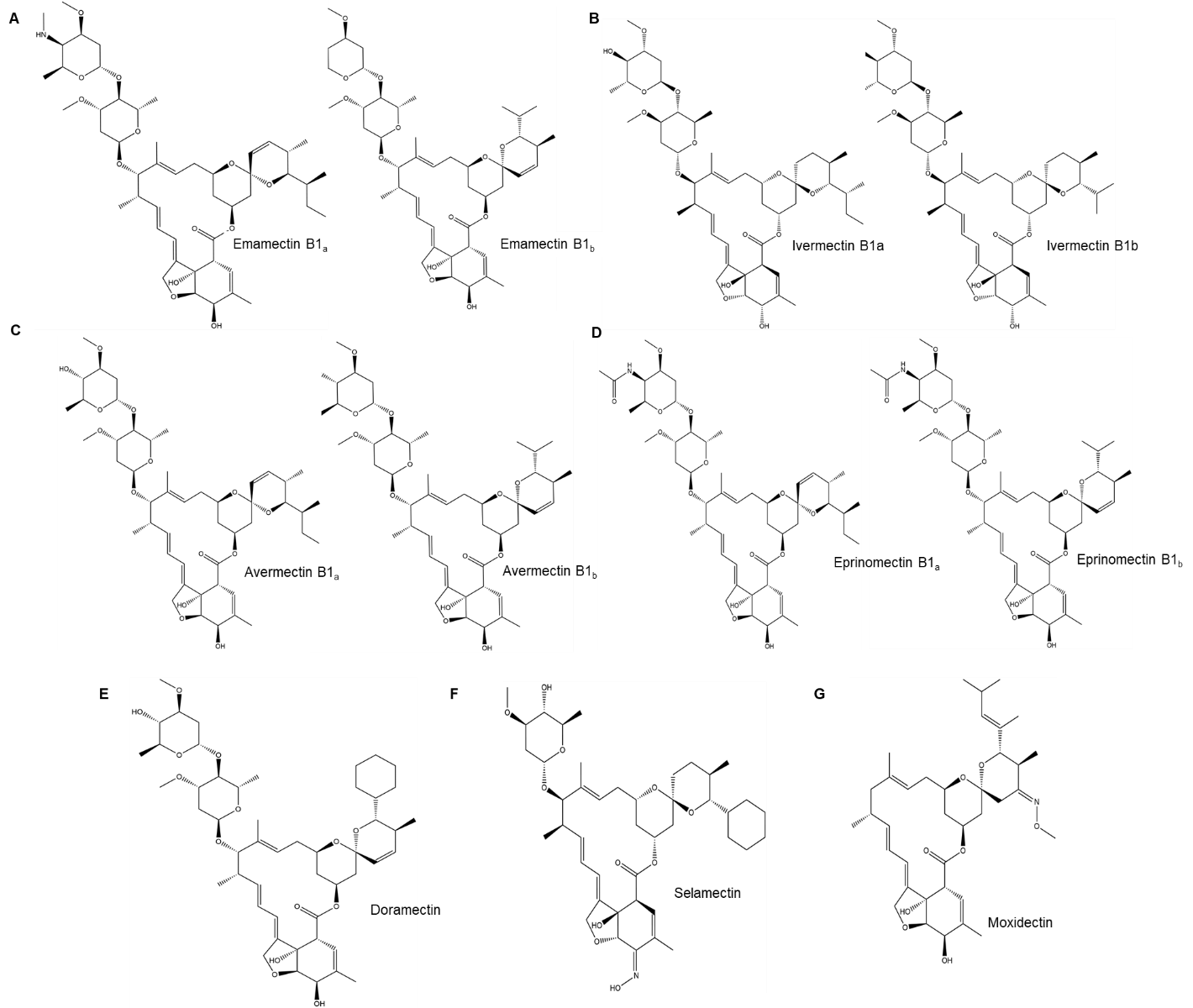


**Fig S2.** Chemical structures of the avermectins and the milbemycin, moxidectin, used in this study. **A)** Emamectin Benzoate (EMB, ratio of ≥90% B1a to ≤10% B1b), **B)** Ivermectin (IVM, ratio of ≥90% B1a to ≤10% B1b), **C)** Avermectin [non-proprietary name, Abamectin (ABM, ratio ≥80% B1a to <20% B1b], **D)** Eprinomectin (EPM, ratio ≥90% B1a to ≤10% B1b), **E)** Doramectin (DRM), **F)** Selamectin (SEL), **G)** Moxidectin (MOX). Chemical structure was drawn with ChemDraw v.20.0 using the following PubChem CIDs: emamectin B1a, 11136686; emamectin B1b, 15279960; ivermectin B1a, 6321424; ivermectin B1b, 6321425; avermectin B1a, 6434889; avermectin b1b, 6858005; eprinomectin B1a, 6444397; eprinomectin B1b, 20055319; doramectin, 9832750; selamectin, 9578507 and moxidectin, 9832912.


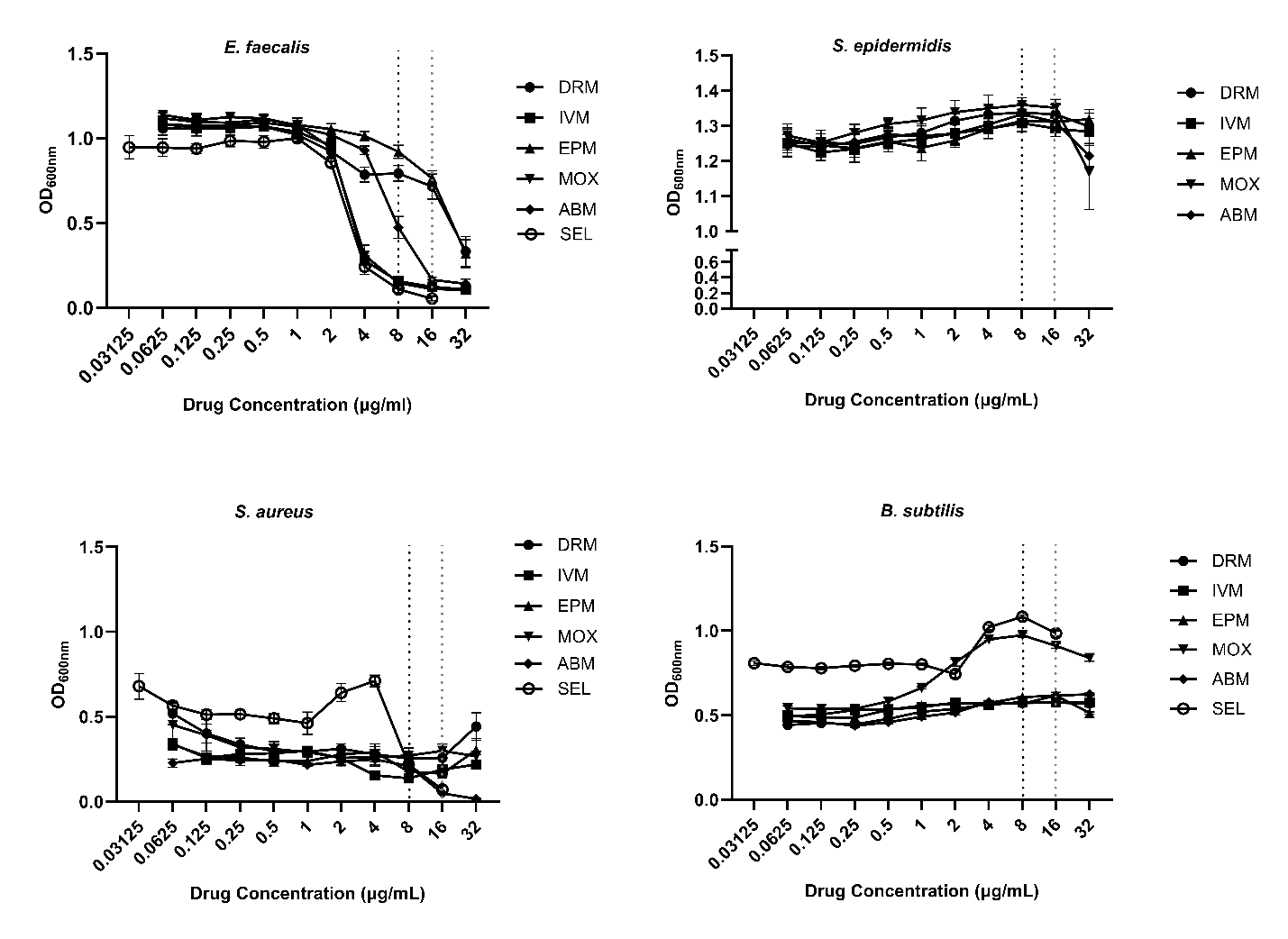


**Fig S3.** Optical densities at 600 nm of the 96-well microtitre susceptibility testing plates taken after 18 hours of incubation at 37°C. Data represents the mean ± SEM of a minimum of three biological replicates, each performed in technical duplicates. DRM, doramectin; IVM, ivermectin; EPM, eprinomectin; MOX, moxidectin; ABM, abamectin; and SEL, selamectin. For DOM, IVM, EPM, MOX and ABM, drug concentrations tested ranged from 0.0625 μg/mL to 32 μg/mL. SEL concentrations tested ranged from 0.03125 μg/mL to 16 μg/mL. The vertical gray dotted line demarcates the highest concentration of SEL, ABM, DRM, EPM and MOX that does not form visible precipitate in MHII broth. The vertical black dotted line demarcates the highest concentration of IVM that does not form visible precipitate in MHII broth. Optical densities at 600 nm were baseline-corrected by subtracting background absorbance of the uninoculated media control from the absorbance of the inoculated media.


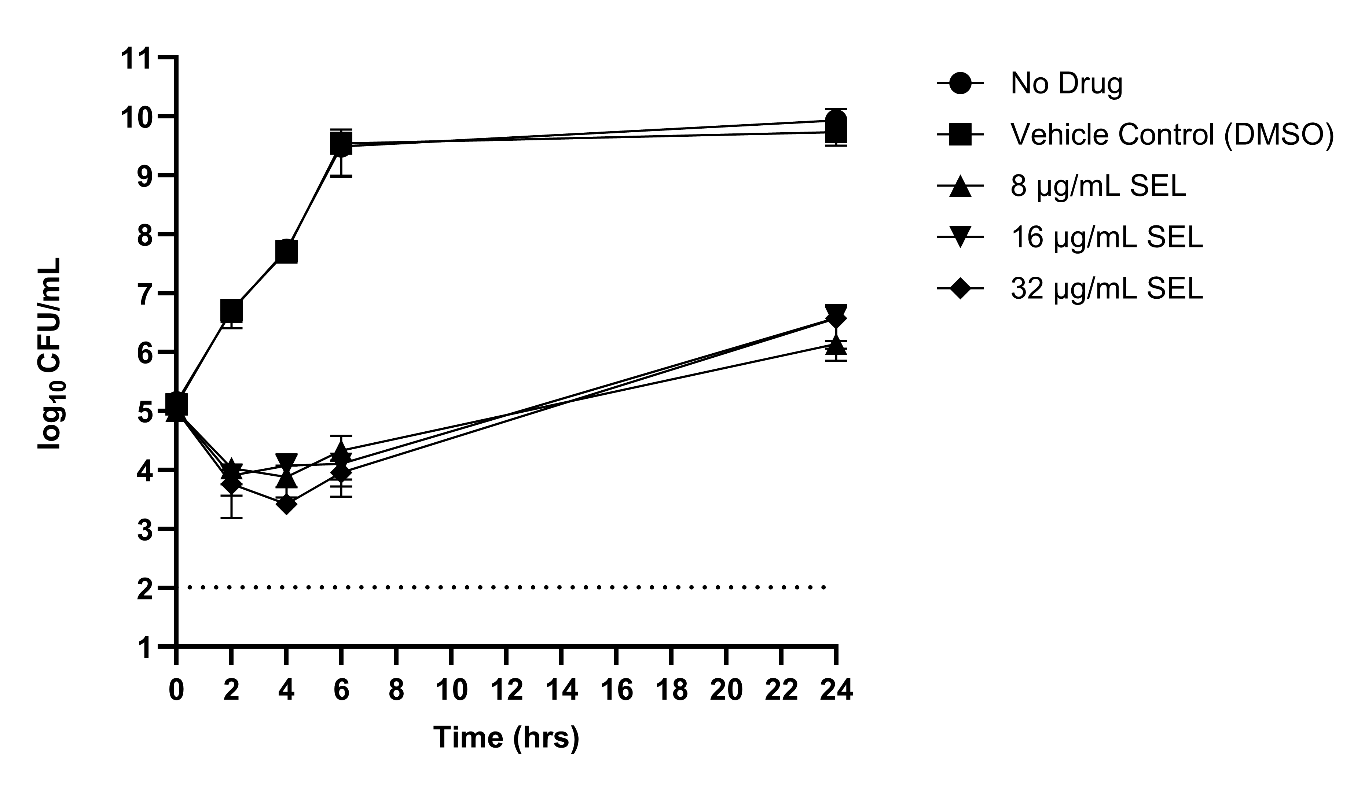


**Fig S4.** SEL is bacteriostatic against *S. aureus* ATCC 25923. Time-kill kinetics of SEL against *S. aureus* was performed in LB broth as outlined by CLSI (1). The dotted black line demarcates a 3-log fold reduction in CFU/mL relative to the initial (t=0) inoculum of the 1 x MIC (16 μg/mL) SEL-treated cells and values above this line indicate bacteriostatic activity.


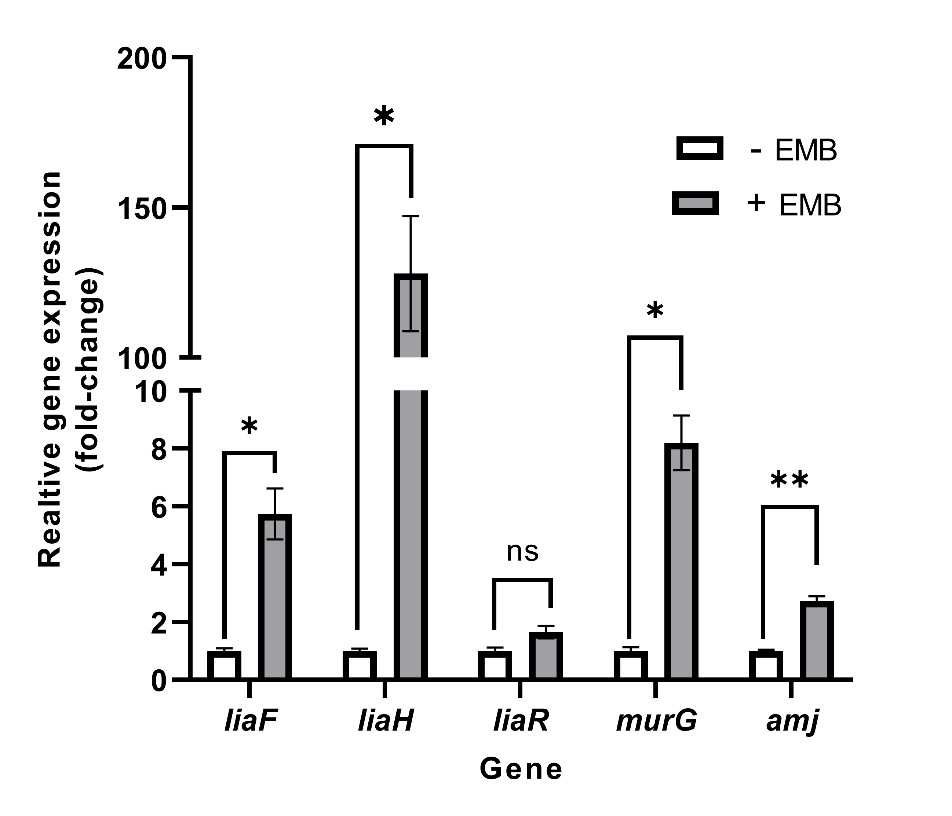


**Fig S5.** Expression of *liaF*, *liaH*, *liaR*, *murG* and *amj* was assessed in log-phase WT *B. subtilis* cells following a 30-minute exposure to 1/4X MIC of EMB (8 μg/mL) using qRT-PCR. In all cases, expression was normalized to *gyrB* and *gatB* and is reported relative to vehicle control (ethanol) treated *B. subtilis* 1A1. Values shown are means ± SEMs from at least three independent determinations performed in technical triplicates. Asterisks indicate a significant difference (* p<0.05, ** p<0.005) by the unpaired t-test with Welch’s correction.


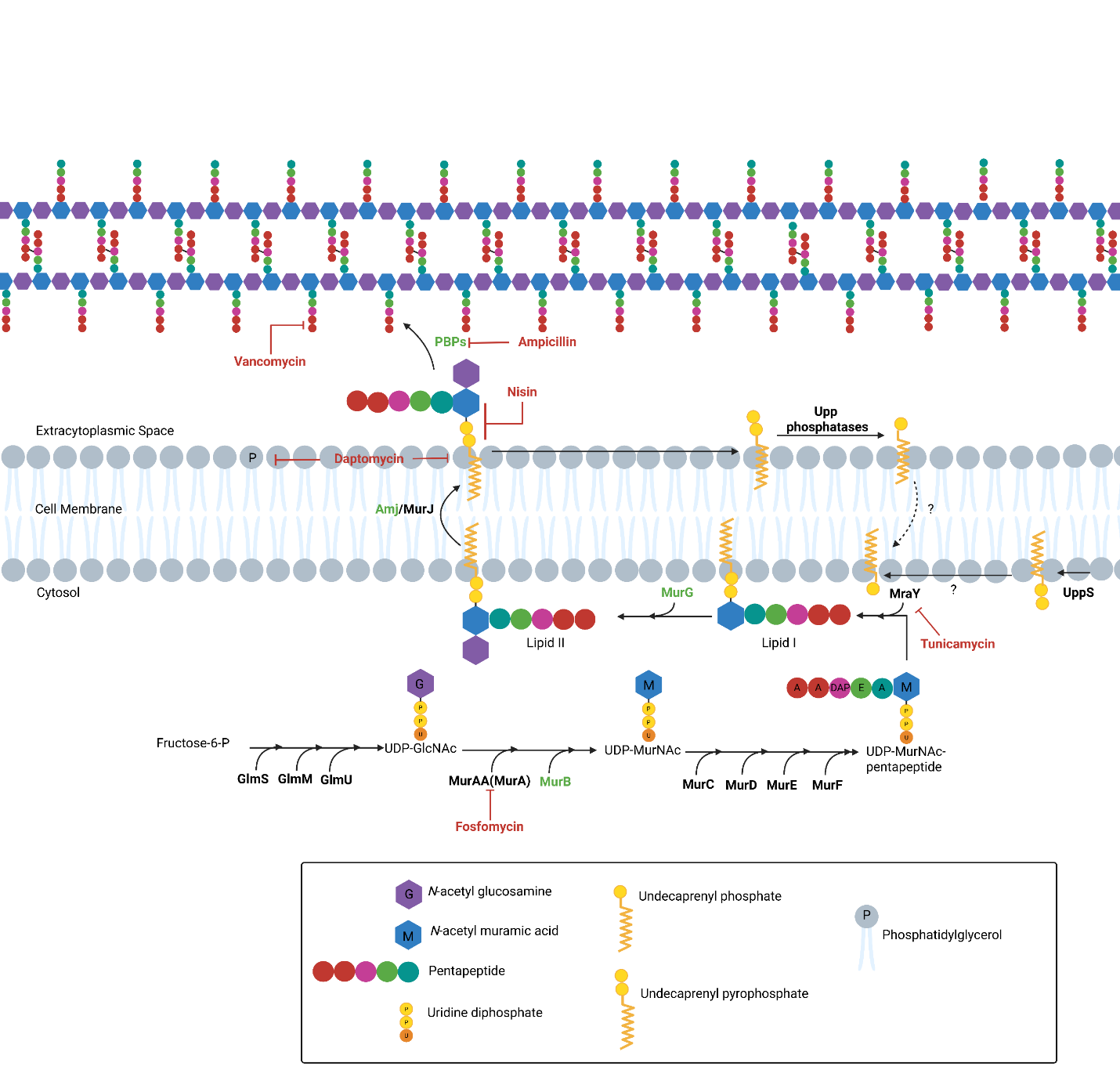


**Fig S6**. Schematic of the peptidoglycan biosynthetic pathway, related to Table 5 and Fig 2. The pathway involves three stages: i) the cytoplasmic steps, which result in the production of uridine diphosphate *N*-acetyl muramic acid pentapeptide (UDP-MurNAc-pentapeptide) ii) the cytoplasmic membrane steps, which involve the lipid carrier, undecaprenyl phosphate and translocation of *N*-acetylglucosaminyl-*N*-acetylmuramyl-(pentapeptide)-diphosphoryl undecaprenol (lipid II) from the inner leaflet to the outer leaflet of the cytoplasmic membrane and iii) extracellular steps, which involve polymerization and crosslinking of the cell wall (2). In the cytoplasmic stage, the enzymes GlmS, GlmM, and GlmU sequentially convert fructose-6-phosphate into uridine diphosphate *N*-acetylglucosamine (UDP-GlcNAc). The enzymes MurA (or MurAA in *B. subtilis*), MurB, and MurC-F sequentially convert UDP-GlcNAc into UDP-MurNAc-pentapeptide (3-5). At the inner leaflet of the cytoplasmic membrane, MraY attaches UDP-MurNAc-pentapeptide to undecaprenyl phosphate to form *N*-acetylmuramyl-(pentapeptide)-diphosphoryl undecaprenol (lipid I). MurG then transfers UDP-GlcNAc to lipid I, yielding lipid II. In *B. subtilis*, the functionally redundant MurJ and Amj (YdaH) flippases translocate lipid II to the outer leaflet of the cytoplasmic membrane, where penicillin binding proteins (PBPs) incorporate peptidoglycan subunits into the growing peptidoglycan layer (59). Following incorporation of peptidoglycan precursor into the cell wall, undecaprenyl pyrophosphate is released, dephosphorylated by the action of Upp phosphatases to form undecaprenyl phosphate, which is translocated back by an unknown mechanism (demarcated by ?) to the inner leaflet of the cytoplasmic membrane for reuse by MraY (6). Enzymes whose genes were upregulated in response to EMB are colored in green front. Peptidoglycan synthesis inhibitors and their corresponding targets are indicated in red font. Bacitracin interacts with the pyrophosphate group of undecaprenyl pyrophosphate (7). Nisin interacts with pyrophosphate group and *N*-acetylmuramic acid moieties of lipid II, and in doing so, forms pores in the membrane, as well as interferes with peptidoglycan synthesis (8). Daptomycin, which selectively disrupts gram-positive bacterial membranes, forms a tripartite complex with lipid II and anionic phospholipid phosphatidylglycerol primarily at the division septum, resulting in the delocalization of peptidoglycan biosynthesis machinery and impeding the incorporation of nascent PG (9, 10). Fosfomycin targets the first committed cytoplasmic step of peptidoglycan precursor biosynthesis by inhibiting MurAA (11, 12). Tunicamycin has two targets; at low concentrations it interferes with the synthesis of wall teichoic acids by inhibiting the TagO enzyme (not shown), and at higher concentrations it inhibits MraY (13-15) (16, 17). Vancomycin interacts with the D-ala-D-ala region of lipid II, interfering with peptidoglycan synthesis (18). Ampicillin blocks assembly of cell wall peptidoglycan by binding to PBPs and inhibiting transpeptidation of the peptide side chains of peptidoglycan units (19). Created in BioRender. Fruci, M. (2025) https://BioRender.com/fz81cb7

**Table S1**. Susceptibility of select gene knockout strains of *B. subtilis* to EMB, where expression of the corresponding genes were induced by EMB.

| **Strain** | **Relevant Genotype** | **Gene expression (Log Fold Change) of EMB-treated cells relative to vehicle control-treated cells^b^** | **EMB MIC (μg/mL)^a^** |
| --- | --- | --- | --- |
| *B. subtilis* 1A1 | WT | - | 32 |
| BKK33090 | Δ*liaS* | 2.628431324 | 32 |
| BKK33080 | Δ*liaR* | NS | 32 |
| BKK33100 | Δ*liaF* | 3.136222349 | 32 |
| BKK33110 | Δ*liaG* | 3.133740407 | 32 |
| BKK33120 | Δ*liaH* | 5.710101363 | 32 |
| BKK33130 | Δ*liaI* | 6.10880438 | 32 |
| BKK04230 | Δ*amj (ydaH)* | 3.879874187 | 32 |
| BKK25000 | Δ*pbpA* | 1.100116449 | 32 |
| BKK34440 | Δ*pbpE* | 2.656799835 | 32 |
| BKK34430 | Δ*racX* | 2.902082692 | 32 |

^a^EMB, emamectin benzoate. For all strains and drugs tested, minimum of three biological replicates were performed in technical duplicate.

^b^Gene expression values were taken from Supplemental File 1.

**References**

1. CLSI. 1999. Methods for determining bactericidal activity of antimicrobial agents. Approved Guideline, CLSI document M26-A. CLSI.

2. Kawakami N, Fujisaki S. 2018. Undecaprenyl phosphate metabolism in Gram-negative and Gram-positive bacteria. *Biosci Biotechnol Biochem* 82:940-946. <https://doi.org/10.1080/09168451.2017.1401915>

3. Barreteau H, Kovač A, Boniface A, Sova M, Gobec S, Blanot D. 2008. Cytoplasmic steps of peptidoglycan biosynthesis. *FEMS Microbiol Rev* 32:168-207. <https://doi.org/10.1111/j.1574-6976.2008.00104.x>

4. Bugg TDH, Braddick D, Dowson CG, Roper DI. 2011. Bacterial cell wall assembly: still an attractive antibacterial target. *Trends Biotechnol* 29:167-173. https://doi.org/10.1016/j.tibtech.2010.12.006

5. Egan AJF, Errington J, Vollmer W. 2020. Regulation of peptidoglycan synthesis and remodelling. *Nat Rev Microbiol* 18:446-460. https://doi.org/10.1038/s41579-020-0366-3

6. Chang HY, Chou CC, Hsu MF, Wang AH. 2014. Proposed carrier lipid-binding site of undecaprenyl pyrophosphate phosphatase from *Escherichia coli*. *J Biol Chem* 289:18719-35. https://doi.org/10.1074/jbc.M114.575076

7. Economou NJ, Cocklin S, Loll PJ. 2013. High-resolution crystal structure reveals molecular details of target recognition by bacitracin. *Proc Nat Acad Sci* 110:14207-14212. [https://doi.org/10.1073/pnas.130826811](https://doi.org/10.1073/pnas.1308268110)

8. Panina I, Krylov N, Nolde D, Efremov R, Chugunov A. 2020. Environmental and dynamic effects explain how nisin captures membrane-bound lipid II. *Sci Rep* 10:8821. https://doi.org/10.1038/s41598-020-65522-y

9. Rimal B, Chang J, Liu C, Rashid R, Singh M, Kim SJ. 2023. The effects of daptomycin on cell wall biosynthesis in *Enterococcal faecalis*. *Sci Rep* 13:12227. https://doi.org/10.1038/s41598-023-39486-8

10. Srivastava D, Patra N. 2024. Elucidating daptomycin’s antibacterial efficacy: insights into the tripartite complex with lipid II and phospholipids in bacterial septum membrane. *J Phys Chem* 128:4414-4427. <https://doi.org/10.1021/acs.jpcb.4c00332>

11. Marquardt JL, Brown ED, Lane WS, Haley TM, Ichikawa Y, Wong C-H, Walsh CT. 1994. Kinetics, stoichiometry, and identification of the reactive thiolate in the inactivation of UDP-GlcNAc enolpyruvoyl transferase by the antibiotic fosfomycin. *Biochem* 33:10646-10651. <https://doi.org/10.1021/bi00201a011>

12. Kahan FM, Kahan JS, Cassidy PJ, Kropp H. 1974. The mechanism of action of fosfomycin (phosphonomycin). *Ann N Y Acad Sci* 235:364-386. <https://doi.org/10.1111/j.1749-6632.1974.tb43277.x>

13. Hancock I, Wiseman G, Baddiley J. 1976. Biosynthesis of the unit that links teichoic acid to the bacterial wall: inhibition by tunicamycin. *FEBS lett* 69:75-80. <https://doi.org/10.1016/0014-5793(76)80657-6>

14. Campbell J, Singh AK, Santa Maria JP, Jr., Kim Y, Brown S, Swoboda JG, Mylonakis E, Wilkinson BJ, Walker S. 2011. Synthetic lethal compound combinations reveal a fundamental connection between wall teichoic acid and peptidoglycan biosyntheses in *Staphylococcus aureus*. *ACS Chem Biol* 6:106-116. <https://doi.org/10.1021/cb100269f>

15. Yoo J, Mashalidis EH, Kuk ACY, Yamamoto K, Kaeser B, Ichikawa S, Lee S-Y. 2018. GlcNAc-1-P-transferase–tunicamycin complex structure reveals basis for inhibition of N-glycosylation. *Nat Struct Mol Biol* 25:217-224. https://doi.org/10.1038/s41594-018-0031-y

16. Brandish PE, Kimura KI, Inukai M, Southgate R, Lonsdale JT, Bugg TD. 1996. Modes of action of tunicamycin, liposidomycin B, and mureidomycin A: inhibition of phospho-N-acetylmuramyl-pentapeptide translocase from *Escherichia coli*. *Antimicrob Agents Chemother* 40:1640-4. <https://doi.org/10.1128/aac.40.7.1640>

17. Tamura G, Sasaki T, Matsuhashi M, Takatsuki A, Yamasaki M. 1976. Tunicamycin inhibits the formation of lipid intermediate in cell-free peptidoglycan synthesis of bacteria. *Agric Biol Chem* 40:447-449. <https://doi.org/10.1080/00021369.1976.10862071>

18. Blaskovich MAT, Hansford KA, Butler MS, Jia Z, Mark AE, Cooper MA. 2018. Developments in glycopeptide antibiotics. *ACS Infect Dis* 4:715-735. <https://doi.org/10.1021/acsinfecdis.7b00258>

19. Nagai K, Davies Todd A, Jacobs Michael R, Appelbaum Peter C. 2002. Effects of amino acid alterations in penicillin-binding proteins (PBPs) 1a, 2b, and 2x on PBP affinities of penicillin, ampicillin, amoxicillin, cefditoren, cefuroxime, cefprozil, and cefaclor in 18 clinical isolates of penicillin-susceptible, -intermediate, and -resistant pneumococci. *Antimicrob Agents Chemother* 46:1273-1280. <https://doi.org/10.1128/aac.46.5.1273-1280.2002>
